# Transcriptional Regulation of Ribosome Components Are Determined by Stress According to Cellular Compartments in *Arabidopsis thaliana*

**DOI:** 10.1371/journal.pone.0028070

**Published:** 2011-12-02

**Authors:** Rodnay Sormani, Céline Masclaux-Daubresse, Françoise Daniele-Vedele, Fabien Chardon

**Affiliations:** Institut Jean-Pierre Bourgin, UMR1318 INRA-AgroParisTech, Saclay Plant Sciences, Versailles, France; University of South Florida College of Medicine, United States of America

## Abstract

Plants have to coordinate eukaryotic ribosomes (cytoribosomes) and prokaryotic ribosomes (plastoribosomes and mitoribosomes) production to balance cellular protein synthesis in response to environmental variations. We identified 429 genes encoding potential ribosomal proteins (RP) in *Arabidopsis thaliana*. Because cytoribosome proteins are encoded by small nuclear gene families, plastid RP by nuclear and plastid genes and mitochondrial RP by nuclear and mitochondrial genes, several transcriptional pathways were attempted to control ribosome amounts. Examining two independent genomic expression datasets, we found two groups of RP genes showing very different and specific expression patterns in response to environmental stress. The first group represents the nuclear genes coding for plastid RP whereas the second group is composed of a subset of cytoribosome genes coding for RP isoforms. By contrast, the other cytoribosome genes and mitochondrial RP genes show less constraint in their response to stress conditions. The two subsets of cytoribosome genes code for different RP isoforms. During stress, the response of the intensively regulated subset leads to dramatic variation in ribosome diversity. Most of RP genes have same promoter structure with two motifs at conserved positions. The stress-response of the nuclear genes coding plastid RP is related with the absence of an interstitial telomere motif known as telo box in their promoters. We proposed a model for the “ribosome code” that influences the ribosome biogenesis by three main transcriptional pathways. The first pathway controls the basal program of cytoribosome and mitoribosome biogenesis. The second pathway involves a subset of cytoRP genes that are co-regulated under stress condition. The third independent pathway is devoted to the control of plastoribosome biosynthesis by regulating both nuclear and plastid genes.

## Introduction

For their survival, cells need to safeguard energy and to adapt their growth and differentiation to the local environmental fluctuations. Ribosomes are integral to the translation of mRNA into proteins and, as such, are considered as housekeeping components of the cells. However, ribosomal biogenesis, as all protein synthesis, is energy-consuming. In yeast, it has been reported that at least 75% of the transcriptional activity is dedicated to ribosome biogenesis [1]. In response to stress such as nutritional limitation, repression of ribosomal protein synthesis has been observed in all kingdoms: bacteria, yeast, animal and plants [2]. Although ribosome proteins are among the most highly conserved proteins across evolution in all kingdoms, the regulatory pathways controlling the genes encoding these proteins remain unclear.

In eukaryotic cells, ribosomes are millions of KDa ribonucleoprotein complexes comprising two subunits, a large and a small subunit, composed of four rRNA together with 48 and 32 ribosomal proteins (RP) respectively. In the model plant *Arabidopsis thaliana*, each cytoplasmic RP (cytoRP) can be encoded by different members of small families including 2 to 7 family members [3], [4]. In the same family, the RP isoforms share between 65% and 100% amino acid sequence identities [3]. To explain the sense of this conserved redundancy, several explanations have been developed, such as a specialization of the role of each member or the need to rapidly regulate the amount of RPs in the cell. There is no evidence for any of these hypotheses in plants [5], [6]. Moreover, mutant analysis of genes encoding cytoRP often shows that several of these genes have specific developmental roles [7].

In plants, growth and development need a fine-tuning in the activity of cytosol, mitochondria and chloroplasts, requiring distinct and accurate regulation of ribosome biogenesis in each compartment. Molecular data and phylogenetic analyses support the cyanobacteria and α-proteobacterial origins of chloroplast and mitochondria, respectively [8]. The prokaryotic gene families encoding the mitochondrial (mitoRP) and plastid RP (plastoRP) are not yet fully listed in Arabidopsis. In a typical prokaryotic organism, such as *Escherichia coli*, 54 RP make up the ribosome [9]. Thus, in chloroplast and mitochondria, 54 RP are expected as in *Escherichia coli*. However, these RP are not encoded only by genes located within the organelle genome. Indeed, many organelle genes including RP ones have been transferred to the nucleus during plants evolution [8]. From genome sequence analyses, Bonen and Calixte [10] identified recently 46 mitochondrial-type RP genes in the nuclear genome and only 7 mitoRP genes in the mitochondria genome of Arabidopsis. Spinach proteomic and genomic analyses showed that the plastoribosome contains 58 plastoRP among which only 22 are encoded by the chloroplast genome [11], [12]. In addition, seven of the nuclear-encoded plastoRP have been categorized as “plant plastid specific” [13], [14]. Experimental evidences also revealed that more complex exchanges exist such as transfer of RP genes from chloroplast to mitochondrion [15].

Ribosome biogenesis is complex and its control depends on both internal and external signaling in all three compartments: cytoplasm, plastid and mitochondrion. Up to now, there is little information on the transcriptional regulation of members within each RP gene family. Recently, Whittle and Krochko [16] analyzed the expression of RP genes in *Brassica napus*, based on RP EST abundances reported in databases. Their results suggest that the differential regulation of the expression level of RP genes within a family is a transcriptional way to adapt ribosome composition in plants. In this study, we first recorded the entire list of genes coding for RPs in Arabidopsis, and the predicted localization for each RP from the literature and sequence genome analysis. By analyzing the corresponding expression pattern of these genes in the CATMA microarray experiment database [17], we revealed that only five different classes of genes exist. These classes of genes defined by their response to environmental stress are related to the predicted localization of the corresponding RP. These results were then validated with a second analysis of another microarray experiment database using the genevestigator tool [18]. We then analyzed the nucleotide motifs in the promoters of each class of RP gene and revealed that *telo*-box motif is absent in the class of nuclear genes encoding plastoRP. Finally, we discussed the global transcriptional regulation of RP genes in plants.

## Results

### The *Arabidopsis* genome contains 429 RP genes

**Figure 1:**
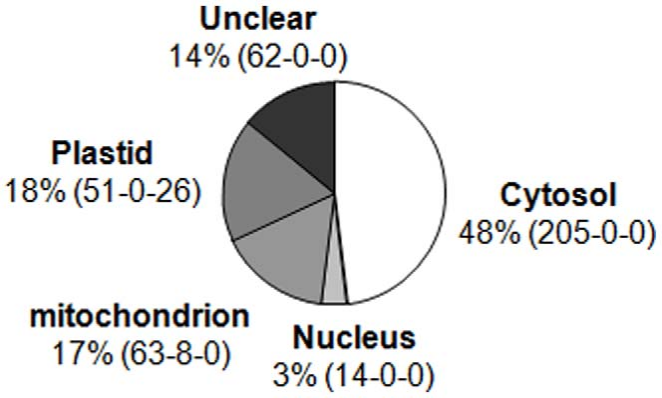
Predicted subcellular localization of RP in Arabidopsis. Subcellular localization of the RP gene product is the result of prediction with SUBA software in accordance to phylogenetic analyses, and with proteomics data when data are available. Full gene list is provided in Table S1. The three numbers in brackets correspond to the gene distribution among the genome localization: nuclear, mitochondrial or plastidial, respectively.

The first step in this study was to obtain a complete list of RP genes in *Arabidopsis thaliana*. For cytoribosome, we used the gene list of cytoRP provided by Barakat *et al.* [3] and data from proteomics studies [4], [19] to perform TBlastN and TBlastX with all the entries available on TAIR 7 Arabidopsis genomics data [20] and to retrieve the missing AGI accession set. In similar manner, for plastoribosome and mitoribosome, the 54 *E. coli* RP [9], available gene list of Arabidopsis mitoRP [10] and proteomic studies of plastoRP in spinach [11], [12], were used to retrieve ribosomal entries in the Arabidopsis genome. In order to discriminate between prokaryotic or eukaryotic origins of each sequence, we performed phylogenic analysis using the *Phylogeny.fr* pipeline [21]. Localizations of the RP were predicted using SUBA software [22], and the predictions were validated when it was possible with available proteomic studies [23], [24], [25], [26], [27], [28], [29]. All these *in silico* analyses provided a list containing 429 references for RP genes with their sub-cellular localization, depicted in Fig. 1 (see Table S1). Among these RP genes, 48% are implicated in cytoRP synthesis. Some new AGI have been identified for cytoRP predicted previously by Barakat et al [3] and additional new cytoRP genes have been found (Table 1). Validation of protein localization for these two gene lists was based on proteomic studies on cytoribosome [4], [19]. For around 3% of the RP genes, RP localization is predicted in the nucleus, reminding us that early steps of cytoribosome biogenesis are nuclear events [30]. MitoRP are encoded by 17% of the RP genes, corresponding to 71 genes distributed in 44 families (see Table S6). Around 18% of the genes have a plastoRP prediction. They correspond to 77 genes distributed in 55 families (see Table S7). 80% of their prediction has been validated by proteomic analyses. The localization remains unclear for 14% of the RP gene list. Among the 429 RP genes, only 19 were annotated as pseudogenes on TAIR 10, with no clear RP localization. Finally, the 429 RP genes are then organized into (i) 221 genes encoding cytoRP (ii) 71 genes encoding mitoRP and (iii) 77 genes encoding plastoRP.

**Table 1:** List of new cytoRP genes coding for cytoRP.

Name	sub-unit	Evolutionary Group	AGI	Identified but remaining undetermined in Barakat et al. [3]	proteomic identifications	
					[19]	[4]
RPL19A	60S	II	AT1G02780	X	X	X
RPL21B	60S	II	AT1G09486	X		
RPL21D	60S	II	AT1G31355	X		
RPL27aC	60S	I	AT1G70600	X	X	X
RPL37aC	60S	III	AT3G60245	X	X	
RPL41C	60S	III	AT2G40205	X		
RPL4C	60S	II	AT2G24730	X		
RPS27C	40S	II	AT3G61111	X		
RPS2B	40S	I	AT1G59359	X	X	
RPS4C	40S	II	AT5G15750	X	X named RPL9D	
RPS7A	40S	III	AT1G48830	X	X	X
RPL10a related	60S	I	AT3G58660			
RPL10aD	60S	I	AT2G27535		X	
RPL18aA	60S	II	AT1G29965		X	
RPL24C	60S	II	AT2G44860		X	
RPL3C	60S	I	AT5G42445			
RPP0D	60S	I	AT1G25260		X	
RPP1D	60S	I	AT5G24510		X	
RPP1D	60S	I	AT3G49460		X	
RPS11D	40S	I	AT2G24110			
RPS15G	40S	I	AT1G33850		X	
RPS25F	40S	III	AT4G34555		X	
RPS29C	40S	I	AT4G33865			
RPS2E	40S	I	AT1G58684		X	
RPS2F	40S	I	AT1G58983		X	

Are provided the protein name, its sub-unit localization, its evolutionary group, its corresponding gene reference number (AGI), if gene has been identified but remaining undetermined in Barakat et al. [3], and proteomic identification from Caroll et al. [19] and Chang et al. [4] when occurring.

### Transcriptional analysis and clustering of RP genes with CATMA database

**Figure 2:**
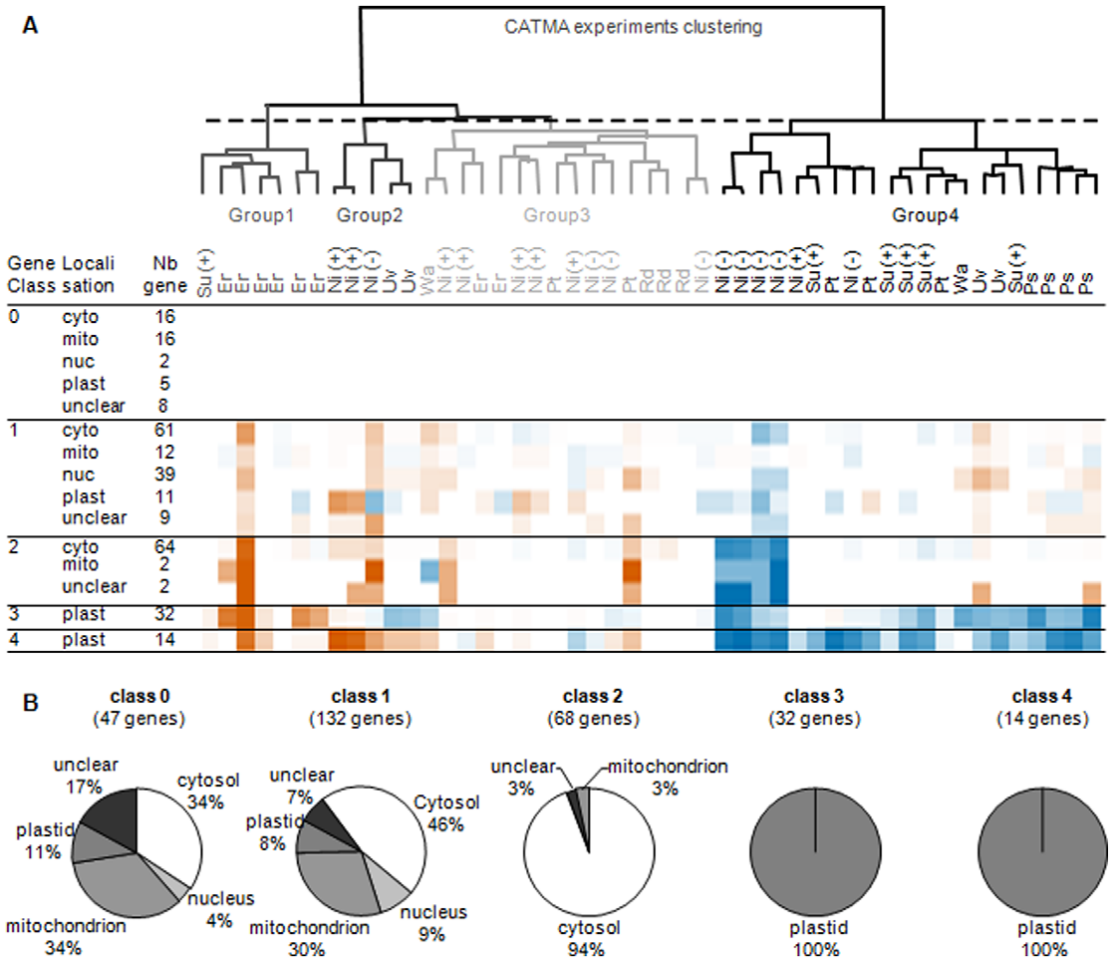
Transcriptional regulation of RP genes is correlated with final localization of the protein. A. Simplified heat map resulting from clustering of 293 probes deriving from RP genes and 49 experiments from CATMA data base (Su(+) for sugar treatment, Er for *Erwinia* attack, Ni(−) and Ni(+) for nitrogen stress and reapplication of nitrogen source after a nitrogen starvation, Uv for UV treatment, Wa for Water stress, Pt for Potyvirus attack, Rd for Rhodococcus attack, Ps for other biotic stress). Scale: blue corresponds to a decreasing ratio of signal between control and treated plants. Red corresponds to an increasing ratio of signal between control and treated plants. Each square represents the average ratio for RP genes with the same location in one class. Individual ratios are provided in Table S2. B. Distributions of predicted localization of RP among the 5 classes depicted above.

To analyze transcriptional regulation of RP genes, dataset from the public CATMA library [17] has been used. From the annotation of nuclear, mitochondrial and plastid genomes, 293 gene-specific probes were retrieved for the 429 RP genes providing expression information on nearly 70% of the RP genes. A set of 49 experiments has been selected for further analysis (list provided in Table S5). These 49 experiments compare WT plant grown in control condition *vs.* treated condition and in the same time show a significant variation in expression for at least one RP gene. Before any computer analysis, all data were homogenized and a code (−1), (+1) and (0) was introduced to indicate that gene was respectively repressed, induced or did not show any variation with significant p-value (as defined in [17]). Two clustering, one for the experiments and another for the genes, have been carried out successively (Fig. 2). Interestingly, CATMA experiments dealing with stress have been clustered into four groups. Groups 1 and 2 include experiments inducing gene expression in response to biotic and abiotic stress, respectively. Group 3 assembles experiments in which stress effect on gene expression was weak. Group 4 includes experiments where plant Carbon/Nitrogen status is affected.

Gene clustering was performed in order to reveal similarities in RP gene expression patterns in response to stress. In the set of CATMA experiments, a list of 47 genes was first identified, showing no significant variation in signal intensity whatever the experiment tested. In this class 0, the different corresponding RP are located within the cytosol, nucleus, mitochondria and plastids with however an over representation of mitoRP (34% of class 0 compared to 17% of the full gene list). Most of the RP pseudogenes belong to this class. From the 246 other genes, a k-means clustering monitored by 4 classes has been used to identify correlations in the regulation of these RP genes. The four classes obtained depict specific transcription patterns in response to environment (Fig. 2A and see Table S2). The most striking result is that these four classes are correlated with the final location of RP. Class 1 represents 132 genes with rare and erratic variations (Fig. 2A). In this group the majority of genes code for cytoRP and mitoRP. MitoRP are over represented, 30% versus 17% of the full RP gene list (Fig. 2B). Class 2 is composed of 68 genes among which 94% are coding for cytoRP. Class 2 is down-regulated by nitrogen starvation and up-regulated by an *Erwinia amylovora* inoculation (Fig. 2A). Classes 3 and 4 are composed of 32 and 14 genes respectively, all of them coding for plastoRP (Fig. 2B). However, in class 3, RP genes are exclusively nuclear whereas in class 4 all genes are plastidial, except two that are nuclear. The two last classes are highly down-regulated by nitrogen starvation and by all the stress affecting the plant Carbon/Nitrogen status (Group 4 of experiment). The partitioning between classes 3 and 4 is based on contrasted responses of RP genes to group 2 experiments, UV stress and nitrogen re-supply (Fig. 2A).

### Validation of the CATMA transcriptional analysis using Affymetrix data available in Genevestigator

**Figure 3:**
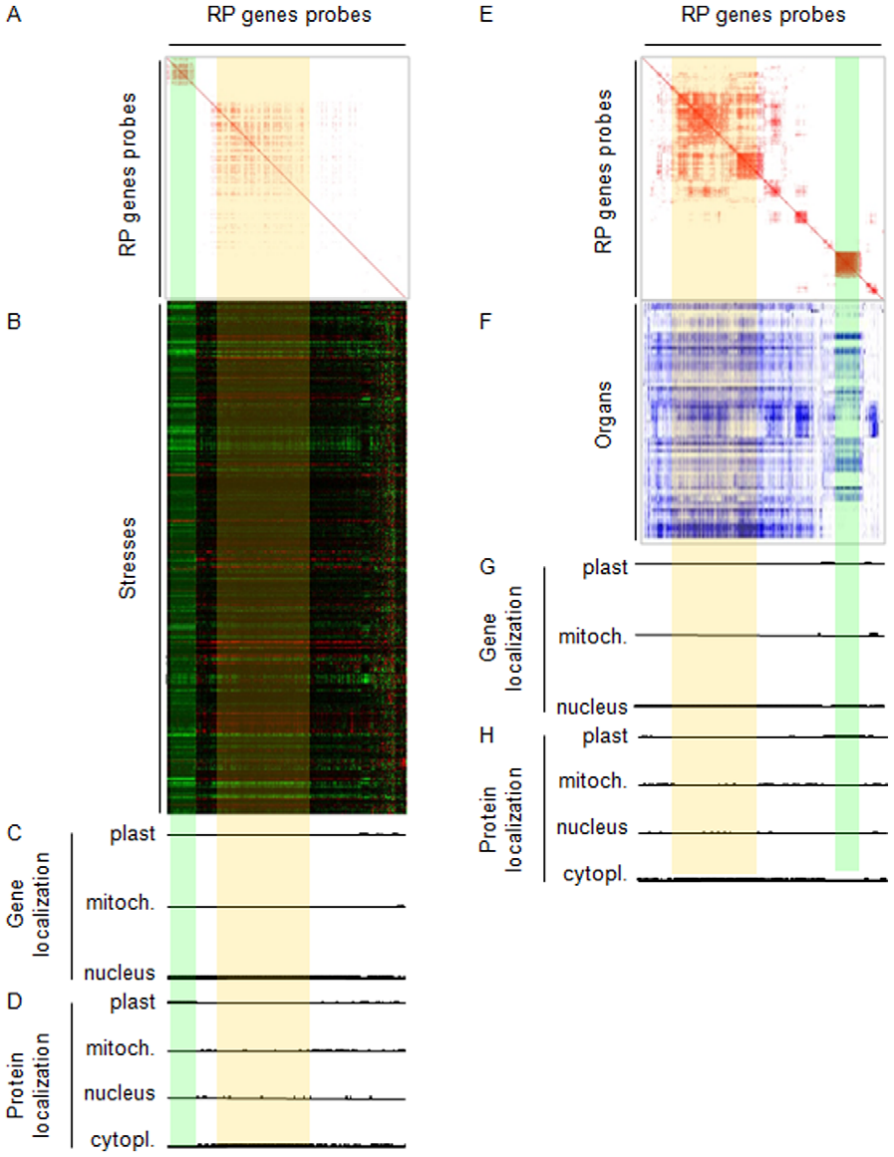
Transcriptional responses of RP genes to abiotic stress in Affymetrix experiments. Transcriptional responses of RP genes to abiotic stresses and their anatomical expressions highlight two correlated group of RP genes in Affymetrix (ATH1 chips) experiments. A. Pearson's correlation matrix of 357 genes in 5747 microarray experiments using data expression results of the conditions series in genevestigator website [18]. Pearson's correlation ≥0.8 are in red. B. Expression profiles used for A results. X and Y axes represent genes and experiments, respectively. C. Protein localization of the gene product from above. D. Gene localization of the RP gene. ABCD are at the same x axis scale. E. Pearson's correlation matrix of 357 genes using data expression results of the Anatomy series of genevestigator, Pearson's correlation ≥0.8 are in red. F. Expression profiles used for E results. X and Y axes represent genes and experiments, respectively. G. Protein localization of gene product from above. H. Localization of RP genes. EFGH are at the same X axis scale. Results of two subgroups of RP genes are highlighted in green and orange in the different panels.

To see if our previous analyses resists to higher complexity, we analyzed a second set of experiment. Results of experiments using Affymetrix ATH1 arrays provide a second and independent dataset that we used to validate our CATMA analysis. Affymetrix results for 5747 experiments have been analyzed using 357 Affymetrix probes available for the 429 RP genes (listed in Table S1). Using Genevestigator [18], hierarchical clustering of gene expression patterns was carried out with Pearson's correlation for the two series of experiments: organ specificity (referred in Genevestigator as anatomy) and stress (referred as conditions) (Fig. 3). Matrix of Pearson's correlations has been done to highlight co-regulated genes in these two different series of experiments. In the two series, two blocks of genes appear with the strongest correlations (*r^2^*≥0.8) (Fig. 3A and 3E). Pattern of co-regulated genes have been underlined in orange and green for the largest and he smallest respectively (Fig. 3B and 3F).

In the stress series, the group of genes underlined in orange is characterized by an important coordinated response to several stimuli (Fig. 3B). Increases in gene expression are observed in light study, circadian clock and shift of etiolated seedling to light experiments. A decrease expression of these genes is observed under osmotic stress, drought, and low light treatments. For the green underlined RP genes, an increase in their expression is observed in response to brassinolide treatment and dramatic reductions are observed during infection with *Pseudomonas syringae*, nitrogen starvation and hypoxia. In the anatomy series of experiments, the RP genes underlined in green are expressed in all green tissue, and their expression is totally excluded from root tissues (Fig. 3F). There is no specific expression pattern among organs for RP genes underlined in orange. In the two series of experiments, most of the RP genes underlined in orange are coding for cytoRP (Fig. 3D and H) with nuclear genome localization (Fig. 3C and G). The RP genes underlined in green are coding for plastoRP (Fig. 3D and H) but are located in the nucleus (Fig. 3C and 3G). Results suggest the hypothesis that RP genes underlined in orange and green are the same in the two Affymetrix series.

### Correspondence between CATMA and Affymetrix co-regulated RP genes

**Figure 4:**
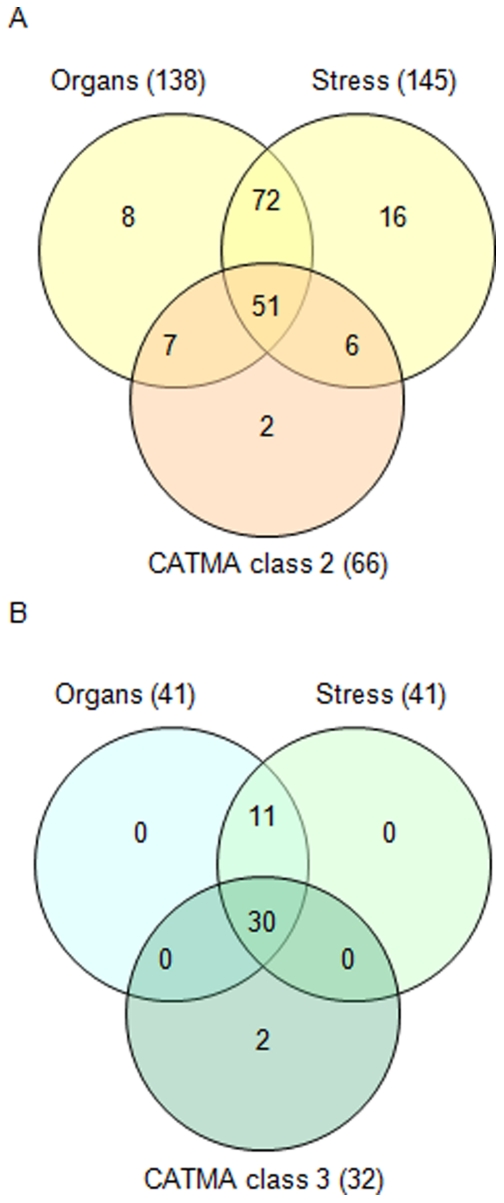
Comparison between Affymetrix and CATMA results. A. Venn diagram showing intersections between sets of genes underlined in orange in the two genevestigator series (anatomy and stimuli) and RP genes in CATMA class 2. B. Venn diagram showing intersections between sets of genes underlined in green in the genevestigator series (anatomy and conditions) and RP genes in CATMA class 3.

In the Affymetrix analysis, RP genes co-regulated and underlined in orange in Fig. 3 set up two groups of 138 and 145 genes for the stress and organ series, respectively. Comparison in a Venn diagram demonstrated that the two groups are predominantly composed of the same genes: among them, 123 (72+51) are common in the two series of experiments (Fig. 4A). All these genes are coding for cytoRP. Comparison of these 123 RP genes to the 66 cytoRP genes belonging to class 2 in CATMA analysis reveals that 51 of them are common between the three lists. Of the 15 remaining CATMA class 2 genes, only two have never been included in any Affymetrix co-regulated groups (underlined in orange in Fig. 3). In contrast, from the 123 RP genes co-regulated in the two Affymetrix series, 72 have not been found in CATMA class 2. However, only half of them have corresponding probes in CATMA arrays.

The lists of RP genes underlined in green in Affymetrix analysis (Fig. 3) have been compared in a same way using Venn diagram. The figure 4B shows that the 41 RP genes selected in the two Affymetrix series are identical, with nuclear genome localization and coding only for plastoRP. Comparison with RP genes in CATMA class 3 shows that 30 of them are common in the three series (Fig. 4B). The two RP genes specific of CATMA class 3 have no Affymetrix corresponding probes. Similarly, among the 11 RP genes specific to the Affymetix series, only two have corresponding CATMA probes and have been found into class 1 (Fig. 2A).

### Two transcriptional pathways regulate cytoRP genes

Each cytoRP can be encoded by different members of small gene families composed of 2 to 7 genes. As shown in two independent data sets (CATMA and Affymetrix), we are able to distinguish a class of cytoRP genes showing highly correlated expression patterns compared to the other cytoRP genes. We then record the number of RP gene families containing members in each transcriptional classes (Table 2). In the CATMA analysis, the 66 class 2 cytoRP genes belong to 44 RP families. Among them, 37 RP gene families have also a member belonging to another transcriptional class, suggesting that 84% of the cytoRP gene families are transcribed following two transcriptional pathways. In the Affymetrix series, the 123 cytoRP genes which are co-regulated belong to 69 families. In these experiments, 70% of the cytoRP gene families are transcribed in two transcriptional pathways. All together, the 51 genes in the intersection of these groups belong to 37 families. Only three of these families exhibit a common transcriptional regulation of their members, suggesting that 92% of these RP families produce cytoRP following two transcriptional pathways.

**Table 2:** Most of the cytoRP gene families were transcribed by two alternative ways.

	Number of genes in class 2	Number of RP gene family containing a class 2 gene	Number of these family containing also another studied gene	Percentage of family having transcriptional alternative to produce RP
CATMA dataset	66	44	37	84%
Affymetrix dataset	123	69	48	70%
Intersection between the two datasets	51	37	34	92%

Results are given for the CATMA dataset, the affymetrix dataset and the intersection between these two sets. Number of genes in class 2 is in the first column. Number of gene families containing class 2 genes is in the second column. The number of gene families containing both genes in class 2 and in other classes is in the third column. The last column is the percentage of family having transcriptional alternative to produce RP obtained by calculating the ratio between third column divided by the second one.

**Figure 5:**
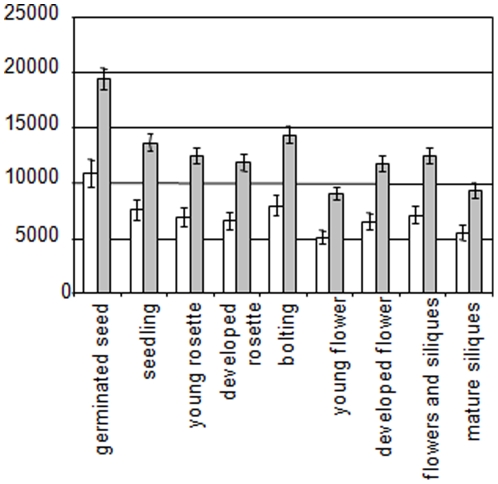
Expression levels of classes 2 and 1 cytoRP genes differ all among the plant development. The class 2 genes have been described as class 2 genes in CATMA and Affymetrix datasets. CytoRP are coded by multigenic families. Each family containing at least one class 2 gene has been considered. The class 1 genes are all the non class 2 cytoRP genes from these families. Values depicted here are means +/− SE. Grey bards correspond to pattern expression of class 2 RP genes. White bards correspond to expression levels of class 1 RP genes.

Finally, despite there is no differential pattern of expression among organs between the co-regulated cytoRP genes and the other cytoRP genes (Fig. 3F), there is a dramatic difference in their level of expression. During all the plant life cycle, the co-regulated cytoRP genes seem to be twice more expressed than the other cytoRP genes (Fig. 5). That could explain why the responses to stress of co-regulated RP genes are so marked.

### Nuclear genes coding for plastoRP have an original pattern of transcriptional regulation

**Figure 6:**
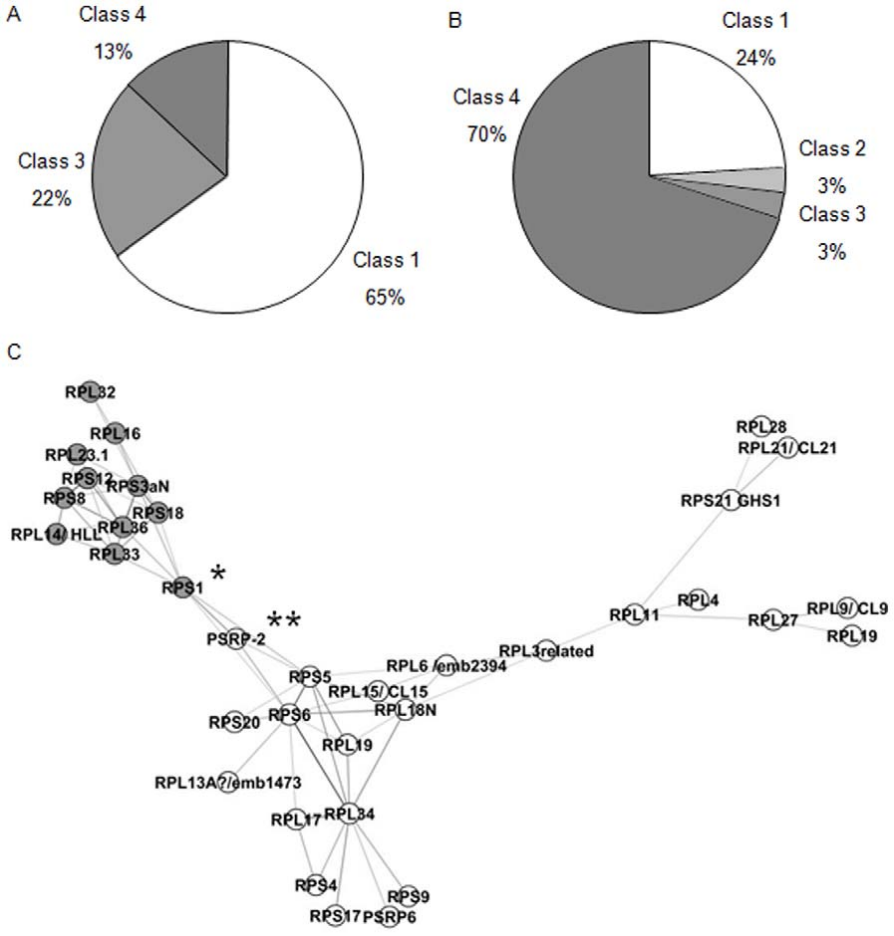
Expression patterns of nuclear and plastidial genes highlight the specificity of nuclear plastoRP gene regulation. A. Distribution of the regulation pattern obtained in CATMA dataset for 46 nuclear genes coding for protein with plastidial location in the 4-means clustering describing four classes of regulation for RP genes. List and heat map are provided in Table S3. B. Distribution of the regulation pattern obtained in CATMA dataset for 30 plastidial genes in the 4-means clustering describing four classes of regulation for RP genes. List and heat map are provided in Table S4. C. PlastoRP gene expressions network in stress conditions. Graphic representation of relationship between plastoRP gene expressions during stress conditions. Genes are represented as nodes, and color and shapes have been assigned according to their localization in nucleus (white) or plastid genome (grey). Edges connecting nodes represent significant correlation between gene expressions in CATMA experiment analyzed. Only correlations upper than 0.75 were kept. Edge thickness is proportional to the correlation. We used the open-source Cytoscape software to visualize the network. (_*_) and (_**_) highlight the key positions of RPS1 and PSRP-2 genes, respectively.

Gene clustering shows that plastoRP genes have a highly specific pattern of expression compared to cytoRP and mitoRP genes (Fig. 2). In particular, the 32 class 3 nuclear genes coding for plastoRP are similarly regulated in response to different stress. Two reasons were investigated to explain such pattern of expression: (i) it was specific to nuclear genes coding for plastoRP or (ii) it was related to the plastid developmental program and then was similar to all the nuclear genes coding for plastidial proteins. To discriminate between these possibilities, 46 nuclear genes coding for other plastidial proteins without any link with ribosome activity have been used for clustering in the same run as RP genes (see Table S3). In the 4-class clustering assay performed on the CATMA dataset, 65% of those genes were grouped within the class 1, 22% in class 3, and 13% in class 4 (Fig. 6A), indicating that the nuclear plastoRP genes of class 3 have a pattern of expression that is specific from any other nuclear encoded plastid components. Similarly, we then wanted to test, if the common expression pattern of the 12 plastidial genes coding for plastoRP was dependent of the plastid development. A list of 29 plastidial genes coding for plastidial proteins without any link with ribosome activity have been used for clustering in the same run as RP genes (see Table S4). In the 4-classes clustering assay made on CATMA dataset, 24% of plastidic genes were grouped within the class 1, 3% in class 2, 3% in class 3, and 70% in class 4 (Fig. 6B). We can conclude that no specific plastidial transcriptional program is dedicated to RP genes, the plastidial genome expression being tightly coordinated through one major transcriptional regulatory pathway.

Regarding the class 3 and class 4 responses to stress (Fig. 2), we tested the correlation of their responses to stress in CATMA dataset. Interestingly, using the open-source Cytoscape software [31], our basic representation of the highest correlations (*r^2^*≥0.75) between gene expressions (Fig. 6C) shows two independent clouds of genes, one for the class 3 and another for the class 4. The nuclear (white) and plastidial (grey) genes from classes 3 and 4 are connected together by two main genes which are *RPS1* and *PSRP2*.

### The pattern of expression of nuclear plastoRP genes is associated to the absence of a motif in their promoters

**Figure 7:**
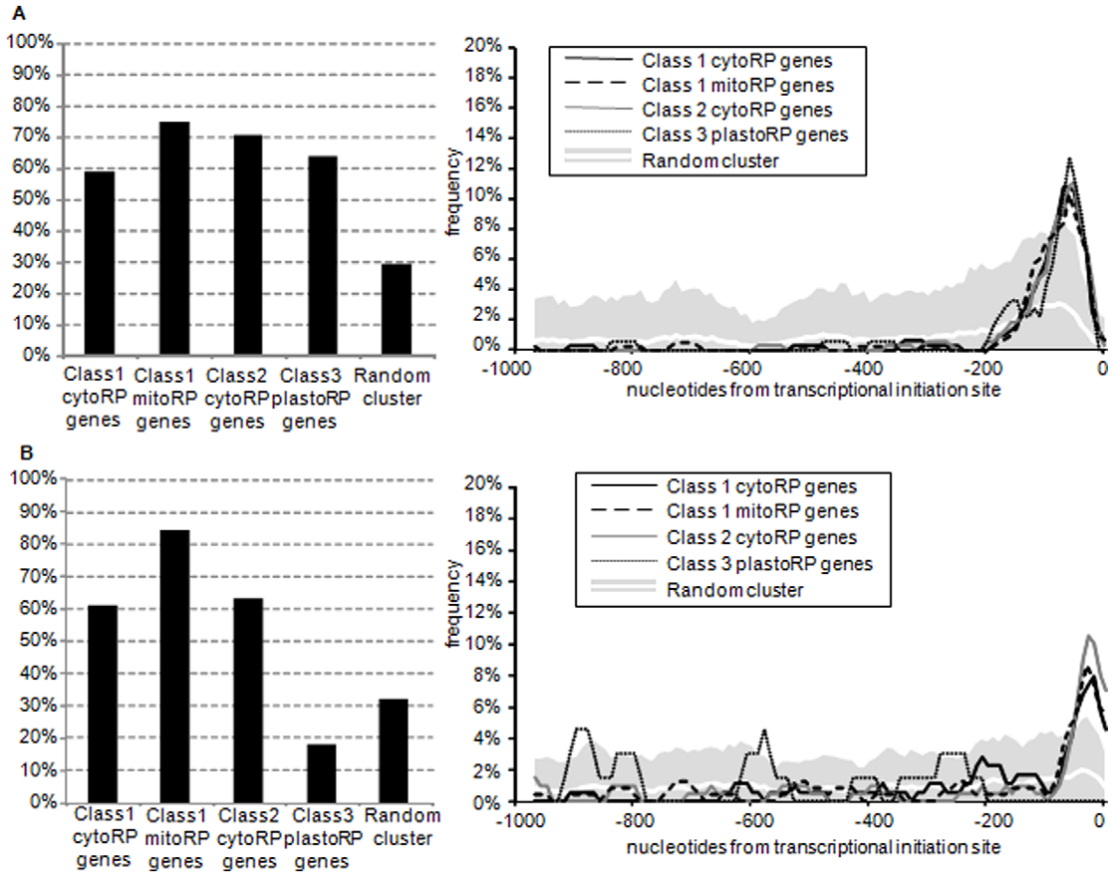
Promoter analyses of site II and telo box motif in RP gene promoters. Promoters of RP genes were scanned to identify two motifs, the site-II motif (A) and the telo box (B). The occurancy of the two motifs are depicted in left panels in regard to the different classes of RP. In right panels the repartition of the selected motifs among the 1000 nucleotides upstream sequence was examined using 50 nucleotides-sliding windows. White line corresponds to the average distribution of random cluster. Grey surface represents the distribution of random cluster, as the 2.5 folds of standard deviation around the mean.

To understand the distribution of expression patterns among RP genes, we analyzed RP promoter sequences of nuclear genes using MEME software to identify specific or common motifs in the classes and FIMO software to find motif occurrences between the classes [32]. Only two interstitial telomere motifs have been found (Fig. 7). The *site II* motif ARGCCCA (R = A or G) [33], [34] is located at −70^+/−25^ nucleotides from the translation initiation site in respectively 59%, 71%, 75% and 64% of the promoters of class 1 cytoRP, class 2 cytoRP, mitoRP and plastoRP genes (Fig. 7A). The *site-II* motif was found in only 29% of random promoter. The *telo-*box motif AAACCCWA (W = A or T) already described in several promoters of RP genes in plants [35] was indeed found in RP promoters but is less represented in promoters of plastoRP genes than in others (Fig. 7B). The *telo*-box is located at −30^+/−25^ nucleotides from the translation initiation site, in respectively 61%, 63% and 84% of promoters for class 1 cytoRP, class 2 cytoRP and class 1 mitoRP (Fig. 7B). Although the *telo*-box motif was found in 32% of random promoter, *Telo*-boxes were never found at this position in the rare plastoRP promoters containing one motif (18%). Trémousaygue et al [36] suggested that the *site-II* motif drives gene expression in actively dividing tissues as young leaves and root primordia, and that the *telo*-box just enhances the expression level. The lack of the *telo*-box enhancer in promoter of nuclear genes encoding plastoRP could explain the difference of expression of these genes in response to environment cues in green tissue (Fig. 3B and 3F).

## Discussion

### Expression program for cytoRP and mitoRP biogenesis is maintained under stressful conditions

This study deals with the regulation of RP encoding genes and we analyzed their expression patterns under stress conditions using two sets of transcriptomic data provided by the CATMAdb resource [17] and genevestigator [18]. The corroborating results reveal that stress does not change dramatically the basal expression program for most of the cytoRP genes and for the nuclear genes coding for mitoRP (Fig. 2 class 0 and 1 and Fig. 3). The existence of such unchanged program for mitoRP is consistent with the fact that this prokaryotic organelle drives energetic function in plant cells. Nevertheless, some stress such as nitrogen starvation lead to a coordinated response of the cytoRP genes (Fig. 2).

The correlated expression of mitoRP and cytoRP nuclear genes certainly needs the action of key transcription factors. The two elements, *telo*-box and *site II* motif, found in most of the promoters of the nuclear genes coding for mitoRP and cytoRP (Fig. 7), could be the cis-elements implied in this co-regulation. Trémousaygue et al. [36] showed that *site II* motif is a target sequence for the transcription factor AtTCP20. The authors revealed that this protein interact with the AtPurα protein, characterized by its ability to interact with the *telo*-box in yeast two hybrids experiments [36], [37]. In addition, *telo*-boxes are also found in genes associated to the cell cycle, such as CYCB1;1 [38]. Plants down-regulated for AtTCP20 gene expression present a strong developmental phenotype, similar to what RP mutants often display [7]. However, only a few RP genes were deregulated in these plants [39].

### Class2 cytoRP genes are dispensable for growth under Carbon/Nitrogen stress conditions

In the budding yeast *Saccharomyces cerevisiae*, two-thirds of cytoRP are encoded by duplicated genes and these paralogous genes are not functionally equivalent suggesting the existence of different populations of ribosomes with diverse functions [40]. Several proteomic analyses suggest that heterogeneity within cytoRP also exists in plants [19], [41].

Among the cytoRP genes we pointed out the class 2 that presented a similar down-regulated expression in response to nitrogen starvation (Fig. 2) and a common response to stress affecting carbon status (Fig. 3B). The cytoRP genes of class 2 belong to small RP gene families with another paralogous gene differentially regulated for at least 70% of them (Table 2). This transcriptional alternative is probably under-estimated by the lack of probes for some RP genes. We hypothesize that there are enough cytoRP family having at least one class 2 RP gene to build up a cytoribosome in class 2 transcriptional ways. The expression of class 2 cytoRP genes decreased dramatically when availability of nitrogen falls whereas other cytoRP genes did not present high variations (Fig. 2. and 3B). The heterogeneity among RP within cytoribosome populations is thus certainly lower under stress conditions. This suggests a specialization of ribosome complex composition in response to stress. The hypothesis of specialization of RP among a family is supported by the recent study of leaf developmental process in Arabidopsis *rp* mutants [42].

### Nuclear genes coding for plastoRP have a specific program of transcription

Interestingly, genes coding for RP of plant organelles do not respond to stress in the same manner if their proteins are targeted to mitochondria or plastids. PlastoRP genes have a highly specific pattern of expression compared to cytoRP and mitoRP genes (Fig. 2, 3B and 3F). Two classes of plastoRP genes differing by their nuclear or plastid genome localization were distinguished based on their contrasted responses to UV stress and nitrogen-resupply conditions (classes 3 and 4 respectively in Fig. 2).

Co-regulation of nuclear genes by the GUN pathway has been clearly demonstrated for plastidial proteins involved in metabolic status of chloroplast [43]. Although our analysis shows that the plastoRP nuclear genes (class 3) are indeed responding to the carbon/nitrogen status of the plant (Fig. 2 and 3B), their expression program appeared RP specific and different from the one of nuclear genes coding for other plastidial proteins (Fig. 6A), suggesting a co-regulation mechanism independent of the GUN pathway. However, the results that we obtained on plastidial genes encoding plastoRP must be carefully analyzed since the polyadenylation of the corresponding mRNAs is a marker step for their degradation in plastids [44]. Because the probes used for CATMA and affymetrix hybridization experiments were synthesized from mRNA using oligo dT primers, the differential expression of RP plastidial genes might have been such biased (Fig. 2 and 3B). In fact, our analysis shows that the down regulation of RP plastidial gene expression under stress is not a specific program but is related to a global plastid RNA steady state level (Fig. 6B), in contrast to nuclear genes coding for plastoRP.

### Cis and trans factors might control the expression of the nuclear genes coding for plastoRP

Proteomic analysis of chloroplast ribosomes had identified in addition to prokaryotic orthologs, seven plant plastid-specific RP (PSRP1-7), encoded by nuclear genes [13], [14]. Yamaguchi and Subramanian [13] proposed that the PSRP form a plastid-specific translation regulatory module and their working model involves PSRP2 interacting with the plastidial encoded RPS1 protein. Interestingly, we found that *RPS1* and *PSRP2* are the two RP genes that connect the nuclear and plastidial classes 3 and 4 in our representation of the highest correlations of gene expressions (Fig. 6C). This finding reinforces the hypothesis of a fundamental role of the PSRP in the regulation of translation in plastid.

In cytoRP promoters of class 1 and class 2 and in mitoRP promoters of class 1, the *site II* motif is located at −70^+/−25^ nucleotides from the transcription initiation site and the *telo*-box is located at −30^+/−25^ nucleotides from the transcription initiation site. Presence of these two elements in RP promoter might allows to a set of transcription factors to coordinate expression as observed for the biogenesis of the plant mitochondrial respiratory chain [45]. Strikingly, the *site II* motif is present at the same localization in the plastoRP promoters than in the other RP promoters, but the structure of plastoRP promoter is not conserved for *telo*-boxes. The absence of the *telo*-box enhancer in promoter of nuclear plastoRP genes could be a part of the mechanism resulting in a more intense response of class 3 genes to environment fluctuations (Fig. 3B). It also might be an indication that the evolutionary processes of plastoRP genes migrating to the nuclear genome did not recruit this motif in contrast to mitoRP genes.

### Conclusion

**Figure 8:**
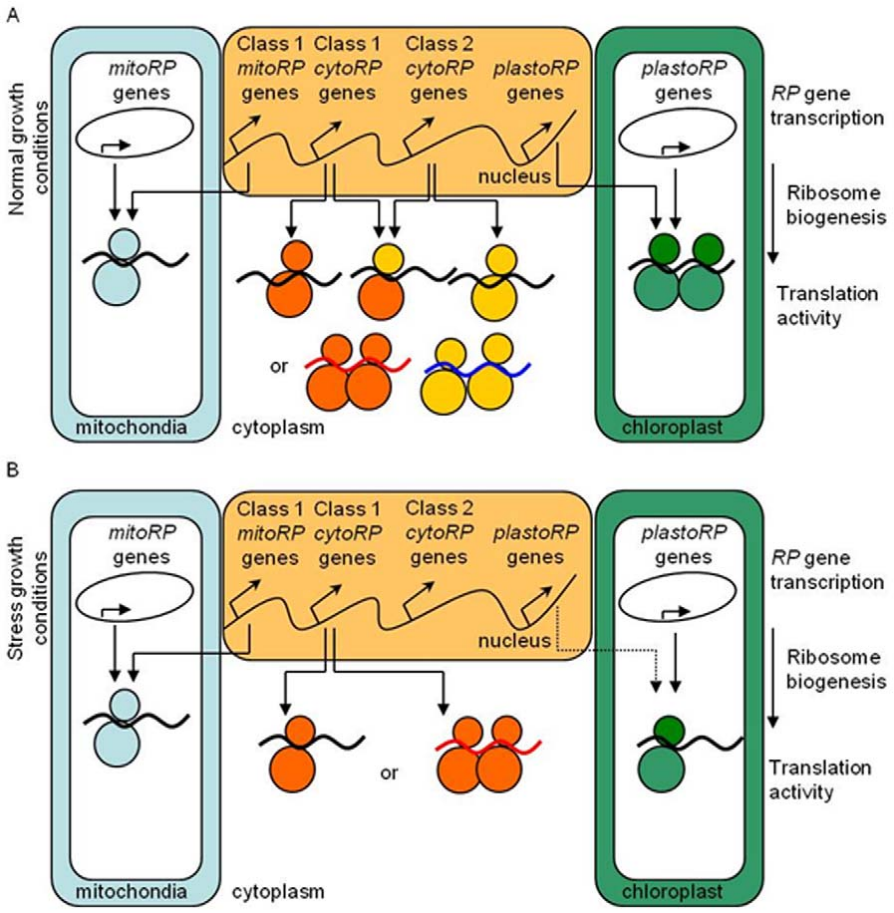
Model of ribosome regulation by stress in plants. A. Ribosomal protein fluxes in cell of plant growing in normal condition. From the nuclear RP genes, three fluxes lead to form ribosomes and to assure high protein synthesis into the cytoplasm, mitochondrions and plastids. The diversity of cytoRP forms allows the cell to produce numerous complexes giving possibilities to produce specialized ribosomes with specialized translational activies. B. In stress condition, cell keeps only class1 RP fluxes which decrease level of translation in cytoplasm and sustains translation in mitochondrions. Cytoplasmic translational activity could be affected globally or only on a subset of mRNA. For stress affecting also plastids, cells dramatically reduce plastoRP synthesis affecting their translational activity.

The regulation of RP protein synthesis could be required to save energy and nutrient and to adapt in the same time to environment. Conversely, phenotypic response to stress could be a consequence of the lack of ribosome needed to translate specific mRNAs. Komili et al. [40] proposed that there is a “ribosome code” that influences translation similarly to the “histone code” that influences transcription. From our results, we propose a model of the ribosome biogenesis by three transcriptional pathways (Fig. 8).

The first pathway controls the housekeeping cytoribosome and mitoribosome biogenesis using key transcriptional regulators as AtTCP20 [36]. The second pathway involves class 2 cytoRP genes that are up-regulated under non-limiting conditions but down-regulated under stress. The translational response to stress might be a general decrease in translation of all mRNAs, as observed in response to dehydration stress or hypoxia [46], [47], or a reduction in translation of a subset of mRNAs, as observed in response to salt or cadmium stress [48], [49]. During plant development, specialization of mRNA translation has been observed in mutants belonging to the same cytoRP family [50], [51] and this evidence supports our last hypothesis. The third independent pathway controls ribosome biosynthesis in plastids by regulating both nuclear and plastid genes. This last pathway is highly sensitive to environmental features and could be convey by plant specific gene coding for plastoRP like PSRP2.

## Methods

### Genes and probes

RP gene names, AGI, and corresponding probes in CATMA and Affymetrix arrays are given in Table S1. From the SUBA database [22], we selected non-RP nuclear genes with protein localization inferred both in plastid by GFP fusions and MS/MS determination. Genes coding for RP were removed from the list, only nuclear genes with CATMA probes were kept. AGI and corresponding probes of non-RP nuclear-genes with protein targeted to plastidial compartment are listed in Table S3. From the gene list available on the CATdb [17], we selected 29 non-RP plastidial genes with specific probes. AGI and corresponding probes of plastidial gene without link to ribosome are presented in Table S4.

### Clustering of CATMA experiments

CATdb (http://urgv.evry.inra.fr/CATdb) has been used to obtained value for hybridization ratios of analyzed genes [17]. Clustering of RP genes have been made using k-means methods monitored by 3 to 5 fixed classes, 50 times each. Class number of each RP gene was determined following the modal value of the 50 clustering results, in the three cases. Evaluation of the distribution of RP genes into 3 to 5 classes indicated that the most efficient class number is four. Only the results of the 4-means clustering are depicted here.

### Clustering of Affymetrix experiments

Hybridizations results using Affymetrix technology were analyzed by using genevestigator software [18]. Clustering tree of RP genes has been done using Pearson distance calculation.

### Online software

Blast against *A. thaliana* genome have been proceed using TAIR resource [20], (http://www.arabidopsis.org/). Phylogenic trees have been reconstructed automatically by using Phylogeny.fr pipeline with the “one click” mode [21], (http://www.phylogeny.fr/). SUBA software has been used to give a prediction of cellular targeting of the RP [22], (http://www.suba.bcs.uwa.edu.au). The characterization of promoter motifs has been done using MEME and FIMO softwares [32], (http://meme.sdsc.edu/meme4_4_0/intro.html). Representation of correlation between gene expression was generated using open-source Cytoscape software [31], (http://www.cytoscape.org/).

## Supporting Information

Table S1 Full list of RP genes in *Arabidopsis thaliana*. Description: For each gene, the gene reference number (AGI), the common name, the CATMA ID corresponding probe, the Affymetrix ID corresponding probe, the predicted localization of the encoded protein and the predicted eukaryote or prokaryote phylogenic origin are indicated.
(PDF)

Table S2 Clustering of transcriptional response to biotic and abiotic stress of genes coding for RP in the CATMA microarray database.
(XLS)

Table S3 List of 46 nuclear genes coding for chloroplast proteins. Description: Gene reference number (AGI), gene description and CATMA ID corresponding probe are provided for each gene.
(PDF)

Table S4 List of 29 plastidic genes coding for chloroplastic proteins. Gene reference number (AGI), protein short name and CATMA ID corresponding probe are provided for each gene.
(PDF)

Table S5 List of CATMA experiment.
(PDF)

Table S6 List of RP genes coding for mitoRP. Are provided gene reference number (AGI), and common gene names, alternative gene name are provided when it exist. Corresponding ID in CATMA and Affymetrix dataset are listed. Bibliographic evidences have been reported in the two last columns (corresponding citation number in Table S1).
(PDF)

Table S7 List of RP genes coding for plastoRP. Are provided gene reference number (AGI), and common gene names, alternative gene name are also provided when it exist. PSRP are plastid-specific RP. Corresponding ID in CATMA and Affymetrix dataset are also listed. Bibliographic localization evidences have been also summarized in the two last columns (corresponding citation number in Table S1).
(PDF)
