# Correction: Transcriptional Regulation of Ribosome Components Are Determined by Stress According to Cellular Compartments in *Arabidopsis thaliana*

**DOI:** 10.1371/annotation/1abb23f1-9b36-4d8d-97ff-abadf2a58880

**Published:** 2012-01-12

**Authors:** Rodnay Sormani, Céline Masclaux-Daubresse, Françoise Daniele-Vedele, Fabien Chardon

There was an error in the name of the third author. The correct name is Françoise Daniel-Vedele. 

The correct citation is: Sormani R, Masclaux-Daubresse C, Daniel-Vedele F, Chardon F (2011) Transcriptional Regulation of Ribosome Components Are Determined by Stress According to Cellular Compartments in Arabidopsis thaliana. PLoS ONE 6(12): e28070. doi:10.1371/journal.pone.0028070

