# Supplementary material for: Transcriptional Regulation of Ribosome Components Are Determined by Stress According to Cellular Compartments in Arabidopsis thaliana
Source: PLoS One. 2011 Dec 2;6(12):e28070. doi: 10.1371/journal.pone.0028070 (PMC3229498; doi:10.1371/journal.pone.0028070)
Supplement: Table S1 — Full list of RP genes in Arabidopsis thaliana. Description: For each gene, the gene reference number (AGI), the common name, the CATMA ID corresponding probe, the Affymetrix ID corresponding probe, the predicted localization of the encoded protein and the predicted eukaryote or prokaryote phylogenic origin are indicated. (PDF) [file pone.0028070.s001.pdf]

| Gene reference number (AGI) | Gene Common Name | Gene model type on TAIR 7 | Phylogeny | Subunit | CATMA ID | Affymetrics ID | Predicted Protein localisation | ms/ms localisation evidences | GFP localisation evidences |
|---|---|---|---|---|---|---|---|---|---|
| AT5G24490 | PSRP-1 | protein coding | | | | 249742_at | plastid | 9, 2 | |
| AT3G52150 | PSRP-2 | protein coding | | | CATMA3A45070 | 252032_at | plastid | 9, 27, 6, 2, 14, 1, 15 | |
| AT1G68590 | PSRP-3 | protein coding | | | | 262283_at | plastid | 9, 2, 14 | |
| AT5G15760 | PSRP-3 | protein coding | | | CATMA5A14030 | 246517_at | plastid | | |
| AT2G38140 | PSRP-4 | protein coding | | | CATMA2A36435 | 267088_at | plastid | 9 | 26 |
| AT3G56910 | PSRP-5 | protein coding | | | | 246294_at | plastid | 9, 2, 14, 1 | |
| AT5G17870 | PSRP-6 | protein coding | | | CATMA5A16150 | 250058_at | plastid | | |
| AT4G29060 | PSRP-7 | protein coding | | | | | plastid | | |
| AT1G08360 | RPL10aA | protein coding | euk | Large | CATMA1A07350 | 261694_at | cytosol | 8, 5 | |
| AT2G27530 | RPL10aB | protein coding | euk | Large | CATMA2A25970 | 266210_at | cytosol | 4, 5 | |
| AT5G22440 | RPL10aC | protein coding | euk | Large | CATMA5A19900 | 249945_at | cytosol | 3, 5 | 3 |
| AT2G42650 | RPL10a related | protein coding | euk | Large | CATMA2A41050 | | nucleus | | |
| AT3G58660 | RPL10a related | protein coding | euk | Large | CATMA3A51670 | 251538_at | nucleus | 3 | 3 |
| AT1G06380 | RPL10a related | protein coding | prok | Large | CATMA1A05420 | 259392_at | plastid | 9, 1 | |
| AT2G42710 | RPL1 | protein coding | prok | Large | CATMA2A41110 | 263975_at | mitochondrion | | |
| AT3G63490 | RPL1 | protein coding | prok | Large | | 251120_at | plastid | 11, 9, 6,13, 2 | |
| AT2G27535 | RPL10aD | protein coding | euk | Large | CATMA2A25980 | | cytosol | | |
| AT1G14320 | RPL10A | protein coding | euk | Large | CATMA1B13325 | 261490_at | cytosol | 7 | |
| AT1G26910 | RPL10B | protein coding | euk | Large | CATMA1A25120 | 263686_at | cytosol | 1 | |
| AT1G66580 | RPL10C | protein coding | euk | Large | CATMA1A55860 | 256385_at | cytosol | 7, 18, 5 | |
| AT5G13510 | RPL10 | protein coding | prok | Large | | 245852_at | plastid | 9, 6 | |
| AT3G12370 | RPL10 | protein coding | prok | Large | CATMA3A11320 | 256234_at | mitochondrion | | |
| AT1G32990 | RPL11 | protein coding | prok | Large | CATMA1A31300 | 261190_at | plastid | 9, 6, 2, 1 | |
| AT4G35490 | RPL11 | protein coding | prok | Large | CATMA4A37150 | 253138_at | mitochondrion | | |
| AT5G51610 | RPL11 | protein coding | prok | Large | CATMA5A47540 | 248414_at | plastid | | |
| AT3G58700 | RPL11B | protein coding | euk | Large | | 251552_at | cytosol | 18 | |
| AT5G45775 | RPL11D | protein coding | euk | Large | CATMA5A41760 | | cytosol | 18 | |
| AT1G70190 | RPL12 | protein coding | prok | Large | CATMA1A59480 | 264702_at | mitochondrion | 19 | |
| AT2G03130 | RPL12 | protein coding | prok | Large | CATMA2A02040 | 266729_at | unclear | | |
| AT3G06040 | RPL12 | protein coding | prok | Large | CATMA3A05100 | 258466_at | unclear | | |
| AT4G36420 | RPL12 | protein coding | prok | Large | CATMA4A38000 | 246210_at | mitochondrion | | |
| AT4G37660 | RPL12 | protein coding | prok | Large | CATMA4A39180 | 253058_at | mitochondrion | | |
| AT2G37190 | RPL12A | protein coding | euk | Large | | 265445_at | cytosol | 3, 11, 4 | |
| AT3G27830 | RPL12A | protein coding | prok | Large | | | plastid | 4, 9, 27,13, 2, 14 | |
| AT3G27840 | RPL12B | protein coding | prok | Large | CATMA3A27650 | 257223_at | plastid | | |
| AT3G53430 | RPL12B | protein coding | euk | Large | CATMA3A46380 | 251938_at | cytosol | 4 | |
| AT3G27850 | RPL12C | protein coding | prok | Large | | 257225_s_at | plastid | 27,13, 2, 14, 1 | |
| AT5G60670 | RPL12C | protein coding | euk | Large | CATMA5A56410 | 247584_at | cytosol | 4 | |
| AT3G01790 | RPL13 | protein coding | prok | Large | CATMA3A00800 | 258995_at | mitochondrion | | |
| AT3G48130 | RPL13 homolog/RSU1 | pseudogene | | Large | CATMA3A41135 | 252347_at | unclear | | |
| AT3G49010 | RPL13B | protein coding | euk | Large | | 252294_at | cytosol | 3, 8, 4, 7, 5 | 3 |
| AT1G78630 | RPL13A | protein coding | prok | Large | CATMA1A67690 | 263131_at | plastid | 9, 6, 2, 1 | |
| AT3G48960 | RPL13C | protein coding | euk | Large | CATMA3A41940 | 252283_at | cytosol | 23 | |
| AT5G23900 | RPL13D | protein coding | euk | Large | CATMA5A21370 | 249815_at | cytosol | 4, 23 | |
| AT3G07110 | RPL13aA | protein coding | euk | Large | | 258837_at | cytosol | 3, 2 | 3 |
| AT3G24830 | RPL13aB | protein coding | euk | Large | CATMA3A24750 | 257599_at | cytosol | 5 | |
| AT4G13170 | RPL13aC | protein coding | euk | Large | CATMA4A13270 | 254763_at | cytosol | | |
| AT5G48760 | RPL13aD | protein coding | euk | Large | | 248655_at | cytosol | | |
| AT1G17560 | RPL14/ HLL | protein coding | prok | Large | CATMA1A16610 | 260683_at | mitochondrion | 15 | |
| ATCG00780 | RPL14/ HLL | protein coding | prok | Large | C165 | 244982_at | plastid | 9 | |
| AT5G46160 | RPL14/ HLP | protein coding | prok | Large | CATMA5A42160 | 248878_at | mitochondrion | | 15 |
| AT2G20450 | RPL14A | protein coding | euk | Large | CATMA2A18940 | 263372_at | cytosol | 17, 4, 18 | |
| AT4G27090 | RPL14B | protein coding | euk | Large | | 253901_at | cytosol | 17, 3, 4, 2, 7, 18, 5 | |
| AT4G23620 | RPL15 | protein coding | prok | Large | CATMA4A25470 | 254228_at | mitochondrion | | |
| AT5G64670 | RPL15 | protein coding | prok | Large | CATMA5A60100 | 247249_at | mitochondrion | | |
| AT3G25920 | RPL15 | protein coding | prok | Large | CATMA3A25740 | 258076_at | plastid | 9, 6, 2 | |
| AT4G16720 | RPL15A | protein coding | euk | Large | | 245342_at | cytosol | 8, 7, 5 | |
| AT4G17390 | RPL15B | protein coding | euk | Large | | 245355_at | cytosol | 3 | |
| ATCG00790 | RPL16 | protein coding | prok | Large | C168 | 244983_at | plastid | 9 | |
| ATMG00080 | RPL16 | protein coding | prok | Large | M015 | | mitochondrion | | |
| AT2G42740 | RPL16A RPL11A? | protein coding | euk | Large | CATMA2A41170 | 263973_at | cytosol | 3, 4, 2, 18 | |
| AT4G18730 | RPL16B RPL11C? | protein coding | euk | Large | CATMA4A19855 | 254617_s_at | cytosol | 18 | |
| AT3G54210 | RPL17 | protein coding | prok | Large | CATMA3A47150 | 251883_at | plastid | 9 | |
| AT5G64650 | RPL17 | protein coding | prok | Large | CATMA5A60080 | 247247_at | mitochondrion | | |
| AT1G27400 | RPL17A | protein coding | euk | Large | CATMA1A25610 | 264438_at | cytosol | 8, 4, 2, 7, 18 | |
| AT1G67430 | RPL17B | protein coding | euk | Large | CATMA1A56750 | 264233_at | cytosol | 3, 4, 18 | |
| AT5G09770 | RPL17family | protein coding | prok | Large | CATMA5A08580 | 250495_at | mitochondrion | | |
| AT3G05590 | RPL18B | protein coding | euk | Large | CATMA3A04615 | 258900_at | cytosol | 22, 8, 4, 2, 7, 18 | |
| AT2G47570 | RPL18A | protein coding | euk | Large | CATMA2A46030 | 245170_at | unclear | | |
| AT5G27850 | RPL18C | protein coding | euk | Large | CATMA5A25280 | 246758_at | cytosol | 4, 18 | |
| AT3G20230 | RPL18N | protein coding | prok | Large | CATMA3A19890 | 257132_at | plastid | | |
| AT3G22450 | RPL18N | protein coding | prok | Large | CATMA3A22450 | 258447_at | mitochondrion | | |
| AT2G28830 | RPL18N | protein coding | | Large | | 266230_at | unclear | | |
| AT5G27820 | RPL18N | protein coding | prok | Large | CATMA5A25250 | 246746_at | unclear | | |
| AT3G45020 | RPL18N | protein coding | prok | Large | CATMA3A38040 | | mitochondrion | | |
| AT1G14205 | RPL18N | protein coding | prok | Large | | | unclear | | |
| AT1G08845 | RPL18N | protein coding | prok | Large | CATMA1A07750 | | mitochondrion | | |
| AT3G17626 | RPL18N | protein coding | prok | Large | | | unclear | | |
| AT5G13720 | RPL18N | protein coding | prok | Large | | 250247_at | plastid | 9, 33 | |
| AT1G48350 | RPL18N | protein coding | prok | Large | CATMA1A39430 | 262235_at | plastid | 9, 1 | |
| AT1G29965 | RPL18aA | protein coding | euk | Large | CATMA1A27980 | | cytosol | | |
| AT1G29970 | RPL18AA | protein coding | euk | Large | CATMA1A27990 | 260026_at | cytosol | | |
| AT2G34480 | RPL18aB | protein coding | euk | Large | CATMA2A32620 | 267007_at | cytosol | 8, 7, 5 | |
| AT3G14600 | RPL18aC | protein coding | euk | Large | CATMA3A13920 | 258090_at | cytosol | 5 | |
| AT1G24240 | RPL19 | protein coding | prok | Large | CATMA1A23130 | 264874_at | mitochondrion | | |
| AT4G11630 | RPL19 | protein coding | prok | Large | | | mitochondrion | | |
| AT5G11750 | RPL19 | protein coding | prok | Large | | | unclear | | |
| AT4G17560 | RPL19 | protein coding | prok | Large | CATMA4A18580 | 245357_at | plastid | 9, 1 | |
| AT5G47190 | RPL19 | protein coding | prok | Large | CATMA5A43180 | 248798_at | plastid | 9, 1 | |
| AT1G02780 | RPL19A | protein coding | euk | Large | CATMA1A01720 | 262117_at | cytosol | 3, 8, 4, 5 | |
| AT3G16780 | RPL19B | protein coding | euk | Large | | 258410_at | cytosol | 8 | |
| AT4G02230 | RPL19C | protein coding | euk | Large | CATMA4A02500 | 255520_at | cytosol | 8, 5 | |
| AT4G16030 | RPL19D | protein coding | | Large | CATMA4A16813 | 245471_at | mitochondrion | | |
| AT2G07715 | RPL2 | protein coding | prok | Large | CATMA2A07160 | | mitochondrion | | |
| AT2G44065 | RPL2 | protein coding | prok | Large | CATMA2A42495 | 267211_at | mitochondrion | | |
| ATMG00560 | RPL2 | protein coding | prok | Large | M164 | 265232_s_at | mitochondrion | | |
| ATMG00690 | RPL2 | protein coding | prok | Large | M273 | 244906_at | mitochondrion | | |
| AT4G14250 | RPL2 | protein coding | prok | Large | CATMA4A14540 | | unclear | | |
| ATCG00830 | RPL2.1 | protein coding | prok | Large | C175 | | plastid | 9 | |
| ATCG01310 | RPL2.2 | protein coding | prok | Large | | 244987_s_at | plastid | | |
| AT1G16740 | RPL20 | protein coding | prok | Large | | 255767_at | mitochondrion | | |

| | | | | | | | | | |
|---|---|---|---|---|---|---|---|---|---|
| ATCG00660 | RPL20 | protein coding | prok | Large | C139 | 244970_at | plastid | 9 | |
| AT1G57860 | RPL21G | protein coding | euk | Large | | 246379_s_at | mitochondrion | | |
| AT1G35680 | RPL21/ CL21 | protein coding | prok | Large | CATMA1A33866 | 262029_at | plastid | 11, 9, 6, 2, 1 | |
| AT4G30930 | RPL21/ NFD1 | protein coding | prok | Large | CATMA4A32590 | 253549_at | mitochondrion | | 29 |
| AT1G09590 | RPL21A | protein coding | euk | Large | | | cytosol | 3, 4, 2 | 3 |
| AT1G09486 | RPL21B | pseudogene | | Large | | | unclear | | |
| AT1G09690 | RPL21C | protein coding | euk | Large | CATMA1A08550 | 264679_s_at | mitochondrion | | |
| AT1G31355 | RPL21D | pseudogene | | Large | | | unclear | | |
| AT1G57660 | RPL21E | protein coding | euk | Large | | | unclear | | |
| AT3G57820 | RPL21F | pseudogene | | Large | | 251604_at | unclear | | |
| AT1G52370 | RPL22 | protein coding | prok | Large | CATMA1A43430 | | mitochondrion | | |
| AT4G28360 | RPL22 | protein coding | prok | Large | CATMA4A30000 | 253773_s_at | mitochondrion | 31 | |
| ATCG00810 | RPL22 | protein coding | prok | Large | | 244985_at | plastid | 9 | |
| AT1G02830 | RPL22A | protein coding | euk | Large | CATMA1A01760 | 262132_at | cytosol | | |
| AT3G05560 | RPL22B | protein coding | euk | Large | CATMA3A04590 | 259112_at | cytosol | 3, 8, 4, 5 | |
| AT5G27770 | RPL22C | protein coding | euk | Large | CATMA5A25180 | 246745_at | cytosol | 3, 8, 4 | |
| AT1G04480 | RPL23A | protein coding | euk | Large | | 263665_at | mitochondrion | | |
| ATCG00840 | RPL23.1 | protein coding | prok | Large | C178 | | plastid | 9 | |
| ATCG01300 | RPL23.2 | protein coding | prok | Large | | 244988_s_at | plastid | | |
| AT3G04400 | RPL23C | protein coding | euk | Large | CATMA3A03370 | 258569_at | mitochondrion | | |
| AT4G39880 | RPL23? | protein coding | prok | Large | CATMA4A41270 | 252850_at | mitochondrion | | |
| AT2G33370 | RPL23B | protein coding | euk | Large | CATMA2A31530 | 255789_at | cytosol | 3, 4 | |
| AT2G39460 | RPL23aA | protein coding | euk | Large | CATMA2A37735 | 266981_at | cytosol | 3, 4 | |
| AT3G55280 | RPL23aB | protein coding | euk | Large | CATMA3A48290 | 251783_at | cytosol | 5 | |
| AT5G23535 | RPL24 | protein coding | prok | Large | CATMA5A21050 | | unclear | | |
| AT5G54600 | RPL24 | protein coding | prok | Large | CATMA5A50455 | 248174_at | plastid | 9 | |
| AT2G44860 | RPL24C | protein coding | euk | Large | CATMA2A43320 | 266822_at | nucleus | 3 | 3 |
| AT2G36620 | RPL24A | protein coding | euk | Large | CATMA2A34850 | 265210_at | cytosol | 8, 4, 2 | |
| AT3G53020 | RPL24B | protein coding | euk | Large | CATMA3A45970 | 251997_at | cytosol | 3 | |
| AT5G66860 | RPL25 | protein coding | prok | Large | CATMA5A62290 | | mitochondrion | 19 | |
| AT3G49910 | RPL26A | protein coding | euk | Large | | 252235_at | cytosol | 3, 11, 8, 4, 2, 7, 5 | 3 |
| AT5G67510 | RPL26B | protein coding | euk | Large | | 247010_at | unclear | | |
| AT2G16930 | RPL27 | protein coding | prok | Large | | 266535_s_at | mitochondrion | | |
| AT5G15220 | RPL27 | protein coding | prok | Large | | | mitochondrion | | |
| AT5G40950 | RPL27 | protein coding | prok | Large | CATMA5A36620 | 249331_at | plastid | 9, 6, 2, 14, 15 | |
| AT5G39800 | RPL27 | protein coding | | Large | | 249424_s_at | mitochondrion | | |
| AT2G32220 | RPL27A | protein coding | euk | Large | CATMA2A30500 | 265730_at | unclear | | |
| AT3G22230 | RPL27B | protein coding | euk | Large | | 256794_at | cytosol | 4, 5 | |
| AT4G15000 | RPL27C | protein coding | euk | Large | CATMA4A15480 | 245372_at | cytosol | 4 | |
| AT1G12960 | RPL27aA | protein coding | euk | Large | | 261200_at | unclear | | |
| AT1G23290 | RPL27aB | protein coding | euk | Large | | 262985_s_at | cytosol | 3, 3 | |
| AT1G70600 | RPL27aC | protein coding | euk | Large | CATMA1A59875 | | cytosol | 4, 5 | |
| AT2G33450 | RPL28 | protein coding | prok | Large | CATMA2A31610 | 255850_at | plastid | 9, | |
| AT1G04457 | RPL28 | pseudogene | | Large | | | unclear | | |
| AT4G31460 | RPL28 | protein coding | prok | Large | CATMA4A33116 | 253538_at | unclear | | |
| AT2G19730 | RPL28A | protein coding | euk | Large | CATMA2A18270 | 266699_at | cytosol | 8, 4 | |
| AT4G29410 | RPL28C | protein coding | euk | Large | CATMA4A31040 | 253728_at | cytosol | 8 | |
| AT1G07830 | RPL29 | protein coding | prok | Large | CATMA1A06860 | 261418_at | mitochondrion | | |
| AT5G65220 | RPL29 | protein coding | prok | Large | CATMA5A60610 | 247201_at | plastid | 9, 2, 1 | |
| AT3G06700 | RPL29A | protein coding | euk | Large | | 258532_at | cytosol | 3 | |
| AT3G06680 | RPL29B | protein coding | euk | Large | | 258521_at | cytosol | | |
| AT3G17465 | RPL3/ RPL3P | protein coding | | Large | CATMA3A16900 | 258404_at | mitochondrion | 19 | |
| AT5G55140 | RPL30 | protein coding | prok | Large | CATMA5A50910 | 248102_at | mitochondrion | | |
| AT1G36240 | RPL30A | protein coding | euk | Large | CATMA1A34350 | 256460_at | cytosol | | |
| AT1G77940 | RPL30B | protein coding | euk | Large | CATMA1A67090 | 262163_at | cytosol | | |
| AT3G18740 | RPL30C | protein coding | euk | Large | CATMA3A18350 | 257753_at | cytosol | 5 | |
| AT1G75350 | RPL31 | protein coding | prok | Large | CATMA1A64680 | 261119_at | plastid | 9, 6, 1 | |
| AT2G19740 | RPL31A | protein coding | euk | Large | CATMA2A18280 | 266700_at | cytosol | 8 | |
| AT4G26230 | RPL31B | protein coding | euk | Large | | 254012_at | cytosol | | |
| AT5G56710 | RPL31C | protein coding | euk | Large | CATMA5A52490 | 247978_at | cytosol | 5 | |
| ATCG01020 | RPL32 | protein coding | prok | Large | C207 | 244960_at | plastid | | |
| AT4G18100 | RPL32A | protein coding | euk | Large | CATMA4A19120 | 254655_s_at | cytosol | 3, 4, 5 | |
| AT5G46430 | RPL32B | protein coding | euk | Large | | | cytosol | 4 | |
| AT3G06320 | RPL33 | protein coding | | Large | CATMA3A05400 | | unclear | | |
| AT5G18790 | RPL33 | protein coding | | Large | | 249975_s_at | unclear | | |
| ATCG00640 | RPL33 | protein coding | prok | Large | C135 | 244968_at | plastid | 9 | |
| AT1G29070 | RPL34 | protein coding | prok | Large | CATMA1A27050 | 260898_at | plastid | | |
| AT1G69620 | RPL34B | protein coding | euk | Large | | 260369_at | cytosol | 4, 7 | |
| AT3G13882 | RPL34 | protein coding | prok | Large | CATMA3A13110 | | unclear | | |
| AT1G26880 | RPL34A | protein coding | euk | Large | CATMA1A25090 | 263691_at | cytosol | 3, 2 | 3 |
| AT3G28900 | RPL34C | protein coding | euk | Large | | 257141_at | cytosol | 5 | |
| AT1G06980 | RPL35aA? | protein coding | euk | Large | CATMA1A06040 | 256066_at | cytosol | 5 | |
| AT1G07070 | RPL35aA | protein coding | euk | Large | CATMA1A06140 | 256065_at | mitochondrion | | |
| AT1G41880 | RPL35aB | protein coding | euk | Large | | 261362_s_at | unclear | 4 | |
| AT1G74270 | RPL35aC | protein coding | euk | Large | CATMA1A63670 | 260258_at | cytosol | 4 | |
| AT3G55750 | RPL35aD | protein coding | euk | Large | | | mitochondrion | | |
| AT3G09500 | RPL35A | protein coding | euk | Large | CATMA3A08360 | 258709_at | cytosol | 3, 4 | |
| AT2G39390 | RPL35B | protein coding | euk | Large | | 266980_at | cytosol | | |
| AT3G55170 | RPL35C | protein coding | euk | Large | | 251834_at | cytosol | | |
| AT5G02610 | RPL35D | protein coding | euk | Large | CATMA5A01700 | 251007_at | cytosol | 7 | |
| AT2G24090 | RPL35family | protein coding | prok | Large | CATMA2A22440 | 266570_at | plastid | 9 | |
| AT5G20180 | RPL36 | protein coding | prok | Large | CATMA5A18620 | 246073_at | unclear | | |
| ATCG00760 | RPL36 | protein coding | prok | Large | C162 | 244980_at | plastid | 9 | |
| AT2G37600 | RPL36A | protein coding | euk | Large | CATMA2A35880 | 267174_at | cytosol | 4 | |
| AT3G53740 | RPL36B | protein coding | euk | Large | CATMA3A46690 | 251926_at | cytosol | 7 | |
| AT5G02450 | RPL36C | protein coding | euk | Large | CATMA5A01530 | 251018_at | cytosol | 4, 7 | |
| AT3G23390 | RPL36aA | protein coding | euk | Large | CATMA3A23320 | 258296_at | cytosol | | |
| AT4G14320 | RPL36aB | protein coding | euk | Large | CATMA4A14640 | 245311_at | cytosol | | |
| AT3G10950 | RPL37aB | protein coding | euk | Large | CATMA3A10000 | 256434_at | cytosol | | |
| AT3G60245 | RPL37aC | protein coding | euk | Large | CATMA3A53270 | 251409_at | cytosol | | |
| AT1G15250 | RPL37A | protein coding | euk | Large | | 262594_at | unclear | | |
| AT2G35180 | RPL37e | pseudogene | | Large | | | unclear | | |
| AT1G52300 | RPL37B | protein coding | euk | Large | CATMA1A43350 | 259612_at | unclear | | |
| AT3G16080 | RPL37C | protein coding | euk | Large | CATMA3A15500 | 258284_at | unclear | | |
| AT2G43460 | RPL38A | protein coding | euk | Large | CATMA2B41860 | 260538_at | cytosol | | |
| AT3G59540 | RPL38B | protein coding | euk | Large | | 251486_at | cytosol | | |
| AT2G25210 | RPL39A | protein coding | euk | Large | | 263585_at | unclear | | |
| AT3G02190 | RPL39B | protein coding | euk | Large | CATMA3A01183 | 259130_at | unclear | | |
| AT4G31981 | RPL39e | protein coding | | Large | | | unclear | | |
| AT4G31985 | RPL39C | protein coding | euk | Large | | 253482_at | unclear | | |
| AT1G43170 | RPL3A | protein coding | euk | Large | CATMA1A36595 | 264421_at | cytosol | 3, 8, 7, 18, 5 | 3 |
| AT1G61580 | RPL3B | protein coding | euk | Large | CATMA1A50680 | 265032_at | cytosol | | |
| AT5G42445 | RPL3C | pseudogene | unclear | Large | CATMA5A38210 | | unclear | | |

| | | | | | | | | | |
|---|---|---|---|---|---|---|---|---|---|
| AT1G58010 | RPL3 | pseudogene | unclear | Large | | | unclear | | |
| AT2G43030 | RPL3related | protein coding | prok | Large | CATMA2A41430 | 265247_at | plastid | 9, 6, 2 | |
| AT1G07320 | RPL4 | protein coding | prok | Large | CATMA1A06385 | 261078_at | plastid | 11, 9, 6,13, 2, 14, 15 | |
| AT3G09630 | RPL4A | protein coding | euk | Large | CATMA3A08490 | 258715_at | cytosol | 3, 8, 4, 2, 18, 5 | |
| AT1G35200 | RPL4B | pseudogene | unclear | Large | | 245782_at | unclear | | |
| AT2G24730 | RPL4C | pseudogene | unclear | Large | CATMA2B23070 | | unclear | | |
| AT5G02870 | RPL4D | protein coding | euk | Large | CATMA5A01950 | 250973_at | cytosol | 3, 8, 4, 2, 7, 18, 5 | 3 |
| AT2G20060 | RPL4 | protein coding | prok | Large | CATMA2A18540 | 265594_at | mitochondrion | | |
| AT3G52590 | RPL40B | protein coding | euk | Large | | 252056_at | cytosol | | |
| AT2G36170 | RPL40A | protein coding | euk | Large | | 263289_at | cytosol | 3 | |
| AT5G40080 | RPL41 | protein coding | prok | Large | | | mitochondrion | | |
| AT2G40205 | RPL41C | protein coding | euk | Large | | | unclear | | |
| AT3G08520 | RPL41D | protein coding | euk | Large | | | unclear | | |
| AT3G11120 | RPL41E | protein coding | euk | Large | | 256438_s_at | unclear | | |
| AT3G56020 | RPL41G | protein coding | euk | Large | | | unclear | | |
| AT2G07725 | RPL5 | protein coding | prok | Large | | | unclear | | |
| ATMG00210 | RPL5 | protein coding | prok | Large | M067 | 266044_s_at | mitochondrion | | |
| AT4G01310 | RPL5 family | protein coding | prok | Large | CATMA4A01510 | 255623_at | plastid | 9, 6, 2, 1 | |
| AT3G59650 | RPL51/S25 | protein coding | | Large | CATMA3A52680 | 251483_at | mitochondrion | | |
| AT3G25520 | RPL5A | protein coding | euk | Large | | 257906_at | cytosol | 22, 8, 4, 2, 18, 5 | 30 |
| AT5G39740 | RPL5B | protein coding | euk | Large | | 249466_at | cytosol | 3, 8, 4 | |
| AT5G40130 | RPL5C | pseudogene | | Large | | 249390_at | unclear | | |
| AT2G18400 | RPL6 | protein coding | prok | Large | | 265338_at | unclear | | |
| AT1G05190 | RPL6 /emb2394 | protein coding | prok | Large | CATMA1A04040 | 264575_at | plastid | 9, 6, 2, 1 | |
| AT1G18540 | RPL6A | protein coding | euk | Large | | 255776_at | cytosol | 17, 8, 2 | |
| AT1G74060 | RPL6B | protein coding | euk | Large | | 260383_s_at | nucleus | 3 | |
| AT1G74050 | RPL6C | protein coding | euk | Large | | | cytosol | 8 | |
| AT4G12600 | RPL7Ae/L30e/S12e/Gadd45 | protein coding | | Large | CATMA4A12760 | 254831_at | cytosol | | |
| AT4G22380 | RPL7Ae/L30e/S12e/Gadd45 | protein coding | | Large | | 254355_at | unclear | 3 | |
| AT5G08180 | RPL7Ae/L30e/S12e/Gadd45 | protein coding | | Large | CATMA4A05770 | 250546_at | nucleus | 3 | 3 |
| AT5G20160 | RPL7Ae/L30e/S12e/Gadd45 | protein coding | | Large | CATMA5A18600 | 246070_at | nucleus | 3 | 3 |
| AT2G47610 | RPL7aA | protein coding | euk | Large | CATMA2A46040 | 245121_at | cytosol | 3, 2, 7, 5 | 3 |
| AT3G62870 | RPL7aB | protein coding | euk | Large | CATMA3A56030 | 251185_at | cytosol | 7 | |
| AT1G80750 | RPL7A | protein coding | euk | Large | CATMA1A69960 | 261911_at | nucleus | 3, 22 | |
| AT2G01250 | RPL7B | protein coding | euk | Large | CATMA2A00310 | 265736_at | cytosol | 8, 2, 7, 18 | |
| AT2G44120 | RPL7C | protein coding | euk | Large | CATMA2A42540 | 267213_at | cytosol | 3, 2, 18 | 3 |
| AT3G13580 | RPL7D | protein coding | euk | Large | CATMA3A12730 | 256648_at | cytosol | 2, 5, 24, 25 | |
| AT4G36130 | RPL8C | protein coding | euk | Large | CATMA4A37790 | | cytosol | 8, 18, 5 | |
| AT3G51190 | RPL8B | protein coding | euk | Large | CATMA3A44190 | 252144_at | cytosol | 5 | |
| AT2G18020 | RPL8A | protein coding | euk | Large | | 265805_s_at | cytosol | 3, 8, 2, 7, 18, 5 | |
| AT5G53070 | RPL9 | protein coding | prok | Large | CATMA5A48970 | 248295_at | mitochondrion | | |
| AT3G44890 | RPL9/ CL9 | protein coding | prok | Large | CATMA3A37910 | 246339_at | plastid | 9, 6, 2 | |
| AT1G33140 | RPL9C | protein coding | euk | Large | | | cytosol | 1, 18 | |
| AT1G33120 | RPL9B | protein coding | euk | Large | | 261620_s_at | cytosol | 3, 8, 2, 7, 18 | 3 |
| AT4G10450 | RPL9D | protein coding | euk | Large | CATMA4A10490 | 254980_at | cytosol | 5 | |
| AT2G40010 | RPP0A | protein coding | euk | Small | CATMA2B38240 | 267349_at | cytosol | | |
| AT3G09200 | RPP0B | protein coding | euk | Small | CATMA3A08070 | 259006_at | cytosol | 3, 11, 8, 4, 2, 10, 5 | |
| AT3G11250 | RPP0C | protein coding | euk | Small | CATMA3A10270 | 256253_at | cytosol | 4, 10, 5 | |
| AT1G25260 | RPP0D | protein coding | euk | Small | CATMA1A23910 | 245639_at | cytosol | | |
| AT1G01100 | RPP1A | protein coding | euk | Small | CATMA1A00080 | 261578_at | cytosol | 8, 10 | |
| AT4G00810 | RPP1B | protein coding | euk | Small | CATMA4A00880 | 255657_at | cytosol | | |
| AT5G47700 | RPP1C | protein coding | euk | Small | CATMA5A43680 | 248768_at | cytosol | 8 | |
| AT5G24510 | RPP1D | protein coding | euk | Small | CATMA5A22110 | 249738_at | cytosol | | |
| AT3G49460 | RPP1D | protein coding | euk | Small | | 252259_at | cytosol | | |
| AT2G27720 | RPP2A | protein coding | euk | Small | | 266258_at | cytosol | 11, 5 | |
| AT2G27710 | RPP2B | protein coding | euk | Small | | 266256_at | cytosol | 3, 11, 8, 5 | |
| AT3G28500 | RPP2C | protein coding | euk | Small | CATMA3A28390 | 256597_at | cytosol | 8, 5 | |
| AT3G44590 | RPP2D | protein coding | euk | Small | CATMA3A37500 | 252643_at | cytosol | | |
| AT5G40040 | RPP2E | protein coding | euk | Small | | 249381_at | cytosol | | |
| AT4G25890 | RPP3A | protein coding | euk | Small | CATMA4A27590 | 254030_at | cytosol | 8, 5 | 26 |
| AT5G57290 | RPP3B | protein coding | euk | Small | | 247900_at | cytosol | 23 | |
| AT1G71720 | RPS1 | protein coding | prok | Small | CATMA1A60920 | 261507_at | plastid | | |
| AT5G30510 | RPS1 | protein coding | prok | Small | CATMA5A27970 | | plastid | 9, 27, 2 | |
| AT3G23700 | RPS1 | protein coding | prok | Small | CATMA3A23650 | 257172_at | plastid | 11, 9, 2 | |
| AT3G13120 | RPS10 | protein coding | prok | Small | CATMA3A12140 | 257190_at | plastid | 9 | |
| AT3G22300 | RPS10 | protein coding | prok | Small | CATMA3A22295 | 258454_at | mitochondrion | | |
| AT4G25740 | RPS10A | protein coding | euk | Small | CATMA4A27430 | 254049_at | cytosol | 4 | |
| AT5G41520 | RPS10B | protein coding | euk | Small | CATMA5A37120 | 249310_at | cytosol | 5 | |
| AT5G52650 | RPS10C | protein coding | euk | Small | CATMA5A48520 | 248331_at | cytosol | 5 | |
| ATCG00750 | RPS11 | protein coding | prok | Small | | 244979_at | plastid | 9 | |
| AT5G23740 | RPS11C | protein coding | euk | Small | CATMA5A21225 | 249795_at | cytosol | 4 | 12 |
| AT2G24110 | RPS11 | pseudogene | euk | Small | CATMA2A22460 | | unclear | | |
| AT1G31817 | RPS11/NFD3 | protein coding | prok | Small | CATMA1A30070 | 246266_at | mitochondrion | | |
| AT4G30800 | RPS11B | protein coding | euk | Small | CATMA4A32410 | 253598_at | cytosol | | |
| AT3G48930 | RPS11A | protein coding | euk | Small | CATMA3A41910 | 252297_at | cytosol | 4 | 26 |
| AT2G07679 | RPS12 | protein coding | prok | Small | | | mitochondrion | | |
| AT2G07675 | RPS12 | protein coding | prok | Small | CATMA2A06470 | | mitochondrion | | |
| ATCG00160 | RPS12 | protein coding | prok | Small | C033 | 244996_at | plastid | 9 | |
| ATCG00905 | RPS12 | protein coding | prok | Small | C193 | | plastid | | |
| ATCG01230 | RPS12 | protein coding | prok | Small | | 244940_at | plastid | | |
| AT1G15930 | RPS12A | protein coding | euk | Small | CATMA1A14990 | 261789_at | cytosol | 4, 5 | |
| AT1G80800 | RPS12B | pseudogene | euk | Small | | | unclear | | |
| AT2G32060 | RPS12C | protein coding | euk | Small | CATMA2A30340 | 265671_at | cytosol | 11, 5 | |
| AT1G77750 | RPS13 | protein coding | prok | Small | CATMA1A66900 | 259678_at | mitochondrion | | 20 |
| AT5G14320 | RPS13 | protein coding | prok | Small | CATMA5A12555 | 250190_at | plastid | 9, 2, 1 | 20 |
| AT3G60770 | RPS13A | protein coding | euk | Small | CATMA3A53760 | 251341_at | cytosol | 3, 4, 2 | 3 |
| AT4G00100 | RPS13B | protein coding | euk | Small | CATMA4A00105 | 255706_at | cytosol | 3, 4 | |
| AT2G34520 | RPS14 | protein coding | prok | Small | CATMA2A32660 | 266955_at | mitochondrion | | |
| AT3G52580 | RPS14C | protein coding | euk | Small | CATMA3A45520 | 252055_at | cytosol | | |
| ATCG00330 | RPS14 | protein coding | prok | Small | C071 | 245005_at | plastid | 9 | |
| AT2G36160 | RPS14A | protein coding | euk | Small | CATMA2A34350 | 263286_at | cytosol | 8, 4, 2, 7, 5 | |
| AT3G11510 | RPS14B | protein coding | euk | Small | CATMA3A10470 | 259239_at | cytosol | 3, 4, 5 | |
| ATCG01120 | RPS15 | protein coding | prok | Small | C230 | 244938_at | plastid | 9, 6 | |
| AT1G15810 | RPS15? | protein coding | prok | Small | | 259505_at | mitochondrion | | |
| AT1G80620 | RPS15? | protein coding | prok | Small | CATMA1A69830 | | mitochondrion | | |
| AT1G04270 | RPS15A | protein coding | euk | Small | CATMA1A03115 | 263667_at | cytosol | 8, 7, 5 | |
| AT5G09490 | RPS15B | protein coding | euk | Small | | 245890_at | cytosol | | |
| AT5G09500 | RPS15C | protein coding | euk | Small | | 245883_at | cytosol | | |
| AT5G09510 | RPS15D | protein coding | euk | Small | CATMA5A08340 | 245886_at | cytosol | 3, 7, 5 | |
| AT5G43640 | RPS15E | protein coding | euk | Small | | 249105_at | cytosol | | |
| AT5G63070 | RPS15F | protein coding | euk | Small | CATMA5A58620 | 247416_at | cytosol | | |
| AT1G33850 | RPS15G | protein coding | euk | Small | | 261995_at | cytosol | | |
| AT1G07770 | RPS15AA | protein coding | euk | Small | | 261416_at | cytosol | 8, 4, 7, 5 | |
| AT2G19720 | RPS15AB | protein coding | euk | Small | CATMA2A18260 | 266684_at | unclear | | |

| | | | | | | | | | |
|---|---|---|---|---|---|---|---|---|---|
| AT2G39590 | RPS15AC | protein coding | euk | Small | | 266972_at | cytosol | | |
| AT3G46040 | RPS15AD | protein coding | euk | Small | CATMA3A39040 | 252566_at | cytosol | 5 | |
| AT4G29430 | RPS15AE | protein coding | euk | Small | CATMA4A31060 | 253726_at | cytosol | 5 | |
| AT5G59850 | RPS15AF | protein coding | | Small | CATMA5A55630 | 247654_at | cytosol | | |
| AT5G56940 | RPS16 | protein coding | prok | Small | CATMA5A52690 | 247935_at | mitochondrion | | 32 |
| ATCG00050 | RPS16 | protein coding | prok | Small | C236 | 245049_at | plastid | | |
| ATCG00065 | RPS16 | protein coding | prok | Small | C140 | 244939_at | plastid | | |
| AT4G34620 | RPS16 | protein coding | prok | Small | CATMA4A36460 | 253201_at | plastid | 9, 1 | 32 |
| AT2G09990 | RPS16A | protein coding | euk | Small | CATMA2A07930 | 263821_s_at | cytosol | 4, 2, 5 | |
| AT3G04230 | RPS16B | protein coding | euk | Small | CATMA3A03180 | 258576_at | cytosol | 3, 2 | |
| AT5G18380 | RPS16C | protein coding | euk | Small | | | cytosol | 23 | |
| AT1G79850 | RPS17 | protein coding | prok | Small | CATMA1A69000 | 260165_at | plastid | 9, 6, 2, 1 | |
| AT3G18880 | RPS17 | protein coding | prok | Small | | 256654_at | unclear | | |
| AT1G49400 | RPS17/emb1129 | protein coding | prok | Small | CATMA1A40520 | 262415_at | unclear | | |
| AT2G04390 | RPS17A | protein coding | euk | Small | | | cytosol | 3, 8, 4 | 3 |
| AT2G05220 | RPS17B | protein coding | euk | Small | CATMA2A04100 | | mitochondrion | | |
| AT3G10610 | RPS17C | protein coding | euk | Small | CATMA3A09650 | 258922_at | mitochondrion | | |
| AT5G04800 | RPS17D | protein coding | euk | Small | | 250862_s_at | mitochondrion | | |
| AT1G07210 | RPS18 | protein coding | prok | Small | | 256043_at | mitochondrion | | |
| ATCG00650 | RPS18 | protein coding | prok | Small | C137 | 244969_at | plastid | 9, 6 | |
| AT1G22780 | RPS18A | protein coding | euk | Small | CATMA1A21826 | 264203_at | cytosol | 8, 4, 7 | |
| AT1G34030 | RPS18B | protein coding | euk | Small | CATMA1A32275 | 255977_at | cytosol | 7 | |
| AT4G09800 | RPS18C | protein coding | euk | Small | CATMA4A09870 | 255000_at | nucleus | | 3 |
| AT5G47320 | RPS19 | protein coding | prok | Small | CATMA5A43275 | 248800_at | mitochondrion | | |
| ATCG00820 | RPS19 | protein coding | prok | Small | | 244986_at | plastid | 9, 6 | |
| AT3G02080 | RPS19A | protein coding | euk | Small | CATMA3A01070 | 258858_at | cytosol | 4 | |
| AT5G15520 | RPS19B | protein coding | euk | Small | CATMA5A13770 | 246538_at | nucleus | 3 | |
| AT5G61170 | RPS19C | protein coding | euk | Small | CATMA5A56820 | 247566_at | cytosol | 4 | |
| AT3G03600 | RPS2 | protein coding | prok | Small | CATMA3A02530 | 259196_at | mitochondrion | | |
| AT1G58380 | RPS2A | protein coding | euk | Small | CATMA1A48500 | 245841_s_at | cytosol | 8, 2 | |
| AT1G59359 | RPS2B | protein coding | euk | Small | | | cytosol | | |
| AT2G41840 | RPS2C | protein coding | euk | Small | CATMA2A40240 | 260497_at | cytosol | 3, 5 | |
| AT3G57490 | RPS2D | protein coding | euk | Small | CATMA3A50490 | 251638_at | cytosol | | |
| AT3G33002 | RPS2p | pseudogene | | Small | | | unclear | | |
| AT1G58684 | RPS2E | protein coding | euk | Small | | | cytosol | | |
| AT1G58983 | RPS2F | protein coding | euk | Small | | | cytosol | | |
| AT3G15190 | RPS20 | protein coding | prok | Small | CATMA3A14550 | 256855_at | plastid | 9, 6, 2 | |
| AT3G45030 | RPS20A | protein coding | euk | Small | | 252601_s_at | cytosol | | |
| AT3G47370 | RPS20B | protein coding | euk | Small | CATMA3A40370 | 252413_at | cytosol | 3, 4, 5 | |
| AT5G62300 | RPS20C | protein coding | euk | Small | | | cytosol | 4 | |
| AT1G69110 | RPS20 | pseudogene | | Small | | | unclear | | |
| AT5G27700 | RPS21C | protein coding | euk | Small | CATMA5A25110 | 246747_at | cytosol | 23 | |
| AT3G27160 | RPS21 GHS1 | protein coding | prok | Small | CATMA3A26960 | 256753_at | plastid | 2 | |
| AT5G63300 | RPS21 GHS1 | protein coding | prok | Small | CATMA5A58880 | | plastid | | |
| AT3G27450 | RPS21A | pseudogene | euk | Small | | 257738_at | unclear | | |
| AT3G53890 | RPS21B | protein coding | euk | Small | | 251921_at | cytosol | 5 | |
| AT3G09680 | RPS23A | protein coding | euk | Small | | 258712_s_at | cytosol | 3, 5 | |
| AT5G02960 | RPS23B | protein coding | euk | Small | CATMA5A02060 | | cytosol | 5 | |
| AT3G04920 | RPS24A | protein coding | euk | Small | | 259090_at | cytosol | 3, 8, 4, 7, 5 | 3 |
| AT5G28060 | RPS24B | protein coding | euk | Small | CATMA5A25640 | 246730_at | cytosol | 5 | |
| AT2G16360 | RPS25A | protein coding | euk | Small | | 263602_at | cytosol | 3, 2, 7 | |
| AT2G21580 | RPS25B | protein coding | euk | Small | CATMA2A20250 | 263519_at | cytosol | 7 | |
| AT3G30740 | RPS25C | pseudogene | | Small | | 256572_at | unclear | | |
| AT4G39200 | RPS25E | protein coding | euk | Small | CATMA4A40630 | 252912_at | cytosol | | |
| AT4G34555 | RPS25F | protein coding | euk | Small | CATMA4A36400 | 253202_at | cytosol | | |
| AT2G40510 | RPS26A | protein coding | euk | Small | | | cytosol | 4, 2 | |
| AT2G40590 | RPS26B | protein coding | euk | Small | | 255819_s_at | cytosol | | |
| AT3G56340 | RPS26C | protein coding | euk | Small | CATMA3A49310 | 251737_at | cytosol | | |
| AT2G45710 | RPS27A | protein coding | euk | Small | CATMA2A44110 | 267507_at | cytosol | 3, 8 | |
| AT3G61110 | RPS27B | protein coding | euk | Small | | 251357_at | cytosol | 5 | |
| AT5G47930 | RPS27D | protein coding | euk | Small | CATMA5A43900 | 248747_at | cytosol | 8 | |
| AT3G61111 | RPS27E | protein coding | euk | Small | | | cytosol | | |
| AT1G23410 | RPS27aA | protein coding | euk | Small | | 263016_at | cytosol | 8 | |
| AT2G47110 | RPS27aB | protein coding | euk | Small | | 266768_s_at | cytosol | | |
| AT3G62250 | RPS27aC | protein coding | euk | Small | | | cytosol | | |
| AT5G64140 | RPS28C | protein coding | euk | Small | | 247267_at | cytosol | 5 | |
| AT3G10090 | RPS28A | protein coding | euk | Small | | 258937_at | cytosol | | |
| AT5G03850 | RPS28B | protein coding | euk | Small | CATMA5A03040 | 250895_at | cytosol | | |
| AT1G16870 | RPS29 | protein coding | prok | Small | CATMA1A15860 | 256106_at | mitochondrion | | |
| AT3G43980 | RPS29A | protein coding | euk | Small | | 252693_s_at | unclear | | |
| AT3G44010 | RPS29B | protein coding | euk | Small | | | unclear | | |
| AT4G33865 | RPS29C | protein coding | euk | Small | | 253291_at | unclear | | |
| ATMG00090 | RPS3 | protein coding | prok | Small | M018 | 244944_s_at | mitochondrion | | |
| AT2G31610 | RPS3A | protein coding | euk | Small | CATMA2A29850 | 263400_s_at | cytosol | 4, 2, 7 | |
| AT5G35530 | RPS3C | protein coding | euk | Small | CATMA5A30710 | 249700_at | nucleus | 3 | 3 |
| AT3G53870 | RPS3B | protein coding | euk | Small | CATMA3A46820 | | cytosol | 4, 5 | |
| ATCG00800 | RPS3aN | protein coding | prok | Small | C171 | 244984_at | plastid | 9, 2 | |
| AT3G04840 | RPS3aA | protein coding | euk | Small | CATMA3A03860 | 259096_at | cytosol | 8, 2, 5 | |
| AT4G34670 | RPS3aB | protein coding | euk | Small | CATMA4A36493 | 253248_at | cytosol | 3, 8, 5 | |
| AT2G19750 | RPS30A | protein coding | euk | Small | CATMA2A18290 | 266705_at | cytosol | | |
| AT4G29390 | RPS30B | protein coding | euk | Small | CATMA4A31020 | 253715_at | nucleus | | 3 |
| AT5G56670 | RPS30C | protein coding | euk | Small | | 247968_at | cytosol | | |
| ATCG00380 | RPS4 | protein coding | prok | Small | C084 | 245009_at | plastid | 9, 6 | |
| ATMG00290 | RPS4 | protein coding | prok | Small | M097 | 266042_s_at | mitochondrion | 31 | |
| AT5G07090 | RPS4B | protein coding | euk | Small | CATMA5A06270 | 250667_at | cytosol | 7 | |
| AT2G17360 | RPS4A | protein coding | euk | Small | CATMA2A16090 | 264849_at | cytosol | 8, 2, 7 | |
| AT5G58420 | RPS4D | protein coding | euk | Small | CATMA5A54180 | 247815_at | cytosol | 3 | |
| AT1G64880 | RPS5 | protein coding | prok | Small | CATMA1A54180 | 262880_at | mitochondrion | 17 | |
| AT2G33800 | RPS5 | protein coding | prok | Small | CATMA2A32000 | 267435_at | plastid | 11, 9, 6, 2, 14, 15 | |
| AT3G11940 | RPS5A | protein coding | euk | Small | | | cytosol | 8, 2 | |
| AT2G37270 | RPS5B | protein coding | euk | Small | | 265963_s_at | cytosol | 8, 7 | |
| AT1G64510 | RPS6 | protein coding | prok | Small | CATMA1A53800 | 261954_at | plastid | 9, 2, 14 | |
| AT3G18760 | RPS6 | protein coding | prok | Small | CATMA3A18370 | 257755_at | unclear | | |
| AT4G31700 | RPS6A | protein coding | euk | Small | CATMA4A33345 | 253487_at | cytosol | 3, 8, 2, 10 | |
| AT5G10360 | RPS6B | protein coding | euk | Small | CATMA5A09125 | 250440_at | cytosol | 8 | |
| AT2G07696 | RPS7 | protein coding | prok | Small | CATMA2A06830 | | mitochondrion | | |
| ATMG00980 | RPS7 | protein coding | prok | Small | M315 | 263502_s_at | mitochondrion | | |
| ATMG01270 | RPS7 | protein coding | prok | Small | | 265238_s_at | mitochondrion | | |
| ATCG01240 | RPS7.1 | protein coding | prok | Small | | 244992_s_at | plastid | 14, 15 | |
| ATCG00900 | RPS7.2 | protein coding | prok | Small | C191 | | plastid | 9, 2, 15 | |
| AT1G48830 | RPS7A | protein coding | euk | Small | CATMA1A39970 | 256143_at | cytosol | 8, 2, 5 | |
| AT3G02560 | RPS7B | protein coding | euk | Small | CATMA3A01530 | 258486_at | cytosol | | |
| AT5G16130 | RPS7C | protein coding | euk | Small | CATMA5A14420 | 246503_at | cytosol | 3, 8 | |
| AT1G16790 | RPS8 | protein coding | prok | Small | CATMA1A15780 | 255759_at | plastid | 9,13, 1 | |

| | | | | | | | | | |
|---|---|---|---|---|---|---|---|---|---|
| ATCG00770 | RPS8 | protein coding | prok | Small | C163 | 244981_at | plastid | 9 | |
| AT5G20290 | RPS8A | protein coding | euk | Small | CATMA5A18740 | 246068_at | nucleus | 3, 8, 2 | 3 |
| AT5G59240 | RPS8B | protein coding | euk | Small | CATMA5A54990 | 247739_at | unclear | | |
| AT5G06360 | RPS8e | protein coding | euk | Small | CATMA5A05570 | 250703_at | cytosol | | |
| AT1G74970 | RPS9 | protein coding | prok | Small | CATMA1A64335 | 262172_at | plastid | 9, 6, 2, 1 | |
| AT3G49080 | RPS9 | protein coding | prok | Small | CATMA3A42090 | 252287_at | mitochondrion | | |
| AT4G12160 | RPS9A | pseudogene | euk | Small | CATMA4A12280 | 254856_at | unclear | | |
| AT5G15200 | RPS9B | protein coding | euk | Small | | 250159_at | cytosol | 3, 2, 7 | |
| AT5G15750 | RPS9D | protein coding | euk | Small | CATMA5A14020 | 246527_at | cytosol | | |
| AT5G39850 | RPS9C | protein coding | euk | Small | | 249427_at | cytosol | | |
| AT1G72370 | RPSAa | protein coding | euk | Small | CATMA1A61605 | 260426_at | cytosol | 11, 8, 2 | 21 |
| AT3G04770 | RPSAb | protein coding | euk | Small | CATMA3A03785 | 258799_at | cytosol | 2 | |
| AT1G47970 | Rpunknown | protein coding | | | CATMA1A39060 | 259617_at | nucleus | | |
| AT3G17160 | Rpunknown | protein coding | | | CATMA3A16570 | 257879_at | nucleus | | |
| AT4G08695 | Rpunknown | pseudogene | | | | | unclear | | |
| AT3G01800 | RRF | protein coding | | | CATMA3A00810 | 258996_at | unclear | 19 | |
| AT3G63190 | RRF | protein coding | | | CATMA3A56380 | | plastid | 28, 9, 14, 15 | |

| Bibliographic Reference number | PMID Reference | Citation |
|---|---|---|
| 1 | 12938931 | Froehlich et al. 2003 |
| 2 | 15028209 | Kleffmann et al. 2004 |
| 3 | 15496452 | Pendle et al. 2005 |
| 4 | 15574830 | Alexandersson et al. 2004 |
| 5 | 15734919 | Chang et al. 2005 |
| 6 | 16207701 | Peltier et al. 2006 |
| 7 | 17151019 | Jaquinod et al. 2007 |
| 8 | 17317660 | Benschop et al. 2007 |
| 9 | 18431481 | Zybailov et al. 2008 |
| 10 | 18686298 | Whiteman et al. 2008 |
| 11 | 14617066 | Bae et al. 2003 |
| 12 | 15141064 | Tian et al. 2004 |
| 13 | 15322131 | Peltier et al. 2004 |
| 14 | 14729914 | Friso et al. 2004 |
| 15 | 11826309 | Peltier et al. 2002 |
| 16 | 11752383 | Skinner et al. 2001 |
| 17 | 16618929 | Dunkley et al. 2006 |
| 18 | 15215502 | Shimaoka et al. 2004 |
| 19 | 14671022 | Heazlewood et al. 2004 |
| 20 | 11919680 | Mollier et al. 2002 |
| 21 | 15610358 | Koroleva et al. 2005 |
| 26 | 10737809 | Cutler et al. 2000 |
| 22 | 14505352 | Calikowski et al. 2003 |
| 23 | 17934214 | Carroll et al. 2008 |
| 24 | 15821981 | Giavalisco et al. 2005 |
| 25 | 17432890 | Mitra et al. 2007 |
| 27 | 16648217 | Giacomelli et al. 2006 |
| 28 | 18433418 | Ströher et al. 2008 |
| 29 | 16698901 | Portereiko et al. 2006 |
| 30 | 12711688 | Mathieu et al. 2003 |
| 31 | 15276431 | Brugière et al. 2004 |
| 32 | 18453549 | Ueda et al. 2008 |
| 33 | 12766230 | Ferro et al. 2003 |
