# Supplementary material for: Transcriptional Regulation of Ribosome Components Are Determined by Stress According to Cellular Compartments in Arabidopsis thaliana
Source: PLoS One. 2011 Dec 2;6(12):e28070. doi: 10.1371/journal.pone.0028070 (PMC3229498; doi:10.1371/journal.pone.0028070)
Supplement: Table S3 — List of 46 nuclear genes coding for chloroplast proteins. Description: Gene reference number (AGI), gene description and CATMA ID corresponding probe are provided for each gene. (PDF) [file pone.0028070.s003.pdf]

Gene reference number (AGI)	Gene description	CATMA ID
AT1G03160	unknown protein	CATMA1A02050
AT1G03475	LIN2 (LESION INITIATION 2); coproporphyrinogen oxidase	CATMA1A02350
AT1G13270	MAP1C (METHIONINE AMINOPEPTIDASE 1B); metalloexopeptidase	CATMA1A12270
AT1G14410	unknown protein	CATMA1A13410
AT1G21640	NADK2; NAD+ kinase	CATMA1A20720
AT1G50320	ATHX; electron transporter/ thiol-disulfide exchange intermediate	CATMA1A41375
AT2G20890	THF1	CATMA2A19490
AT2G21330	fructose-bisphosphate aldolase	CATMA2A20020
AT2G28900	protein translocase/ protein transporter	CATMA2A27290
AT2G36250	FTSZ2-1; GTP binding / GTPase/ structural molecule	CATMA2A34435
AT2G45300	3-phosphoshikimate 1-carboxyvinyltransferase	CATMA2A43700
AT3G01500	CA1 (CARBONIC ANHYDRASE 1); carbonate dehydratase/ zinc ion binding	CATMA3A00490
AT3G06510	SFR2 (SENSITIVE TO FREEZING 2); hydrolase, hydrolyzing O-glycosyl compounds	CATMA3A05650
AT3G22960	pyruvate kinase	CATMA3A22910
AT3G25690	CHUP1 (CHLOROPLAST UNUSUAL POSITIONING 1)	CATMA3A25470
AT3G26570	PHT2;1	CATMA3A26362
AT3G47070	unknown protein	CATMA3A40150
AT3G49680	branched-chain-amino-acid transaminase/ catalytic	CATMA3A42713
AT3G58010	unknown protein	CATMA3A51010
AT4G00370	ANTR2; organic anion transporter	CATMA4B00440
AT4G15560	CLA1 (CLOROPLASTOS ALTERADOS 1)	CATMA4A16296
AT4G26500	EMB1374; transcription regulator	CATMA4A28070
AT4G30580	ATS2; 1-acylglycerol-3-phosphate O-acyltransferase/ acyltransferase	CATMA4A32190
AT4G31780	MGD1 (MONOGALACTOSYLDIACYLGLYCEROL SYNTHASE 1); 1,2-diacylglycerol 3-beta-galactosyltransferase	CATMA4A33420
AT4G31990	ASP5 (ASPARTATE AMINOTRANSFERASE 5)	CATMA4A33665
AT4G32770	VTE1 (VITAMIN E DEFICIENT 1)	CATMA4A34500
AT4G34240	ALDH3I1; aldehyde dehydrogenase	CATMA4A36060
AT4G35770	SEN1 (DARK INDUCIBLE 1)	CATMA4A37425
AT4G36810	GGPS1 (GERANYLGERANYL PYROPHOSPHATE SYNTHASE 1); farnesyltranstransferase	CATMA4A38380
AT4G37000	ACD2 (ACCELERATED CELL DEATH 2)	CATMA4A38580
AT5G19750	unknown protein	CATMA5A18170
AT5G35220	EGY1 (ETHYLENE-DEPENDENT GRAVITROPISM-DEFICIENT AND YELLOW-GREEN 1); sterol regulatory element-binding protein site 2 protease	CATMA5A30360
AT5G49030	ATP binding / isoleucine-tRNA ligase/ tRNA ligase	CATMA5A44970
AT5G49940	NFU2	CATMA5A45850
AT5G52920	pyruvate kinase	CATMA5A48800
AT5G55280	FTSZ1-1; GTP binding / GTPase/ structural molecule	CATMA5A51023
AT5G57850	4-amino-4-deoxychorismate lyase/ catalytic	CATMA5A53600
AT3G02730	electron transporter/ thiol-disulfide exchange intermediate	CATMA3A01685
AT4G04770	ATABC1	CATMA4A05320
AT4G38970	fructose-bisphosphate aldolase	CATMA4A40410
AT5G17170	electron transporter/ metal ion binding / protein binding	CATMA5A15460
AT5G51070	ERD1 (EARLY RESPONSIVE TO DEHYDRATION 1); ATP binding / ATPase/ nucleoside-triphosphatase/ nucleotide binding / protein binding	CATMA5A46995
AT2G26670	HY1 (HEME OXYGENASE 1)	CATMA2A24995
AT4G27440	PORB (PROTOCHLOROPHYLLIDE OXIDOREDUCTASE B); oxidoreductase/ protochlorophyllide reductase	CATMA4A29015
AT4G35630	PSAT; phosphoserine transaminase/ transaminase	CATMA4A37266
AT5G17520	RCP1 (ROOT CAP 1)	CATMA5A15800
