# Supplementary material for: Transcriptional Regulation of Ribosome Components Are Determined by Stress According to Cellular Compartments in Arabidopsis thaliana
Source: PLoS One. 2011 Dec 2;6(12):e28070. doi: 10.1371/journal.pone.0028070 (PMC3229498; doi:10.1371/journal.pone.0028070)
Supplement: Table S4 — List of 29 plastidic genes coding for chloroplastic proteins. Gene reference number (AGI), protein short name and CATMA ID corresponding probe are provided for each gene. (PDF) [file pone.0028070.s004.pdf]

Gene reference number (AGI)	Protein short name	CATMA ID probe
ATCG00020	PSBA	C002
ATCG00070	PSBK	C015
ATCG00080	PSBI tRNA-Ser	C016
ATCG00120	ATPA	C022
ATCG00210	YCF6	C050
ATCG00220	PSBM	C052
ATCG00270	PSBD	C062
ATCG00280	PSBC	C066
ATCG00300	YCF9	C068
ATCG00350	PSAA	C076
ATCG00420	NDHJ	C093
ATCG00440	NDHC	C097
ATCG00500	ACCD	C110
ATCG00510	PSAI	C115
ATCG00520	YCF4	C116
ATCG00530	YCF10	C118
ATCG00540	PETA	C121
ATCG00590	ORF31	C130
ATCG00600	PETG tRNA-Trp	C131
ATCG00630	PSAJ	C133
ATCG00680	PSBB	C147
ATCG00740	RPOA	C159
ATCG01010	NDHF	C203
ATCG01040	YCF5	C210
ATCG01050	NDHD	C212
ATCG01070	NDHE	C216
ATCG01080	NDHG	C218
ATCG01090	NDHI	C220
ATCG01110	NDHH	C227
