# Supplementary material for: Transcriptional Regulation of Ribosome Components Are Determined by Stress According to Cellular Compartments in Arabidopsis thaliana
Source: PLoS One. 2011 Dec 2;6(12):e28070. doi: 10.1371/journal.pone.0028070 (PMC3229498; doi:10.1371/journal.pone.0028070)
Supplement: Table S5 — List of CATMA experiment. (PDF) [file pone.0028070.s005.pdf]

Type	Experiment	Project name	Title	Pubmed Accession	Organ	Class	Exp Num
Senescence	RIL045LNG 2 / RIL045LN 2	RA04-08_Senescence	Transcriptomic analysis of leaf senescence in Arabidopsis thaliana.	17098848	Rosette leaf	1	1
Erwinia	B14-06h-mutant / B09-06h-WT	RA05-02_Erwinia	Regulation of the Arabidopsis transcriptome by the type three secretion system (TTSS) of Erwinia amylovora		Rosette leaf	1	2
Erwinia	B16-24h-mutant / B11-24h-WT	RA05-02_Erwinia	Regulation of the Arabidopsis transcriptome by the type three secretion system (TTSS) of Erwinia amylovora		Rosette leaf	1	3
DspA	Ea dspA_E 24h set2 / Ea wt 24h set2	RA06-03_DspA-Erwinia	Regulation of the Arabidopsis transcriptome by the type three effector DspA/E of Erwinia amylovora.		Rosette leaf	1	4
DspA	Ea dspA_E 6h set1 / Ea wt 6h set1	RA06-03_DspA-Erwinia	Regulation of the Arabidopsis transcriptome by the type three effector DspA/E of Erwinia amylovora.		Rosette leaf	1	5
DspA	Ea sec 6h set1 / Ea wt 6h set1	RA06-03_DspA-Erwinia	Regulation of the Arabidopsis transcriptome by the type three effector DspA/E of Erwinia amylovora.		Rosette leaf	1	6
DspA	Ea sec 6h set2 / Ea wt 6h set2	RA06-03_DspA-Erwinia	Regulation of the Arabidopsis transcriptome by the type three effector DspA/E of Erwinia amylovora.		Rosette leaf	1	7
Nitrate	F_C10j+1h Arabido / F_C10j Arabido	GNP3-B4_Nitrogen_starvation	Improving the nitrogen use efficiency : from model plant to crop species.		Rosette leaf	2	8
Nitrate	F_C10j+1h Arabido / F_C10j Arabido	GNP3-B4_Nitrogen_starvation	Improving the nitrogen use efficiency : from model plant to crop species.		Rosette leaf	2	9
TOR	col 100(u)M / col 10mM	RA03-07_TOR	Transcriptome analysis of TOR (Target of Rapamycin) mutant.		Roots	2	10
UV	WS+UV 1 / WS 1	AF09_lignin	Lignin mutants and UV stress		Stem	2	11
UV	WS+UV 2 / WS 2	AF09_lignin	Lignin mutants and UV stress		Stem	2	12
Waterstress	35S HAHB4 stressed / 35S HAHB4	RS04-09_hahb4	Identification of targets of the transcription factor hahb-4 in relation to abiotic stress	16972869	Rosette leaf	3	13
Seed dormancy	C50DSb / C10DSb	RA04-05_NO3-seed-dormancy	Nitrate control of Arabidopsis seed dormancy	19074630	Seeds	3	14
Seed dormancy	sil50mM / sil10mM	RA04-05_NO3-seed-dormancy	Nitrate control of Arabidopsis seed dormancy	19074630	Seeds	3	15
Erwinia	C14-06h-mutant / C09-06h-WT	RA05-02_Erwinia	Regulation of the Arabidopsis transcriptome by the type three secretion system (TTSS) of Erwinia amylovora		Rosette leaf	3	16
DspA	Ea dspA_E 6h set2 / Ea wt 6h set2	RA06-03_DspA-Erwinia	Regulation of the Arabidopsis transcriptome by the type three effector DspA/E of Erwinia amylovora.		Rosette leaf	3	17
Nitrate	R_C10j+24h Arabido / R_C10j Arabido	GNP3-B4_Nitrogen_starvation	Improving the nitrogen use efficiency : from model plant to crop species.		Roots	3	18
Nitrate	R_C10j+24h Arabido / R_C10j Arabido	GNP3-B4_Nitrogen_starvation	Improving the nitrogen use efficiency : from model plant to crop species.		Roots	3	19
Potyvirus	Col_LMV_2 / Col_Mock_2	RA03-02_Potyvirus	Transcriptional analysis of Arabidopsis thaliana infected by a potyvirus.		Rosette leaf	3	20
Seed dormancy	c50mM-ds / c10mM-ds	RA04-05_NO3-seed-dormancy	Nitrate control of Arabidopsis seed dormancy	19074630	Seeds	3	21
Nitrate	R_C10j Arabido / R_T0 Arabido	GNP3-B4_Nitrogen_starvation	Improving the nitrogen use efficiency : from model plant to crop species.		Roots	3	22
Nitrate	R_C10j Arabido / R_T0 Arabido	GNP3-B4_Nitrogen_starvation	Improving the nitrogen use efficiency : from model plant to crop species.		Roots	3	23
Potyvirus	CvI_LMV_1 / CvI_Mock_1	RA03-02_Potyvirus	Transcriptional analysis of Arabidopsis thaliana infected by a potyvirus.		Rosette leaf	3	24
Rhodococcus	14.D188.(1,2) / 14.D5.(1,2)	AU05-05_Rhodococcus	Molecular analysis of symptom development on Arabidopsis Thaliana by the pathogenic bacterium Rhodococcus fascians.	19118125	Steam	3	25
Rhodococcus	24.D188.(1,2) / 24.D5.(1,2)	AU05-05_Rhodococcus	Molecular analysis of symptom development on Arabidopsis Thaliana by the pathogenic bacterium Rhodococcus fascians.	19118125	Steam	3	26
Rhodococcus	24.D5.(1,2) / 14.D5.(1,2)	AU05-05_Rhodococcus	Molecular analysis of symptom development on Arabidopsis Thaliana by the pathogenic bacterium Rhodococcus fascians.	19118125	Steam	3	27
Nitrate	R_C2j Arabido / R_T0 Arabido	GNP3-B4_Nitrogen_starvation	Improving the nitrogen use efficiency : from model plant to crop species.		Roots	3	28
Nitrate	F_C10j Arabido / F_T0 Arabido	GNP3-B4_Nitrogen_starvation	Improving the nitrogen use efficiency : from model plant to crop species.		Rosette leaf	4	29
Nitrate	F_C10j Arabido / F_T0 Arabido	GNP3-B4_Nitrogen_starvation	Improving the nitrogen use efficiency : from model plant to crop species.		Rosette leaf	4	30
Nitrate	F_C2j Arabido / F_T0 Arabido	GNP3-B4_Nitrogen_starvation	Improving the nitrogen use efficiency : from model plant to crop species.		Rosette leaf	4	31
Nitrate	F_C2j Arabido / F_T0 Arabido	GNP3-B4_Nitrogen_starvation	Improving the nitrogen use efficiency : from model plant to crop species.		Rosette leaf	4	32
Nitrate	R_C10j+1h Arabido / R_C10j Arabido	GNP3-B4_Nitrogen_starvation	Improving the nitrogen use efficiency : from model plant to crop species.		Roots	4	33
Senescence	RIL232LNG 1 / RIL232LN 1	RA04-08_Senescence	Transcriptomic analysis of leaf senescence in Arabidopsis thaliana.	17098848	Rosette leaf	4	34
Potyvirus	CvI_LMV_2 / CvI_Mock_2	RA03-02_Potyvirus	Transcriptional analysis of Arabidopsis thaliana infected by a potyvirus.		Rosette leaf	4	35
Nitrate	R_C2j Arabido / R_T0 Arabido	GNP3-B4_Nitrogen_starvation	Improving the nitrogen use efficiency : from model plant to crop species.		Roots	4	36
Potyvirus	Bl-1_LMV_2 / Bl-1_Mock_2	RA03-02_Potyvirus	Transcriptional analysis of Arabidopsis thaliana infected by a potyvirus.		Rosette leaf	4	37
Senescence	RIL232LNG 2 / RIL232LN 2	RA04-08_Senescence	Transcriptomic analysis of leaf senescence in Arabidopsis thaliana.	17098848	Rosette leaf	4	38
Senescence	RIL310LNG 1 / RIL310LN 1	RA04-08_Senescence	Transcriptomic analysis of leaf senescence in Arabidopsis thaliana.	17098848	Rosette leaf	4	39
Senescence	RIL310LNG 2 / RIL310LN 2	RA04-08_Senescence	Transcriptomic analysis of leaf senescence in Arabidopsis thaliana.	17098848	Rosette leaf	4	40
Potyvirus	Bl-1_LMV_1 / Bl-1_Mock_1	RA03-02_Potyvirus	Transcriptional analysis of Arabidopsis thaliana infected by a potyvirus.		Rosette leaf	4	41
Waterstress	Col0 Stressed / Col0	RS04-09_hahb4	Identification of targets of the transcription factor hahb-4 in relation to abiotic stress	16972869	Rosette leaf	4	42
UV	EBL87+UV 1 / EBL87 1	AF09_lignin	Lignin mutants and UV stress		Stem	4	43
UV	EBL87+UV 2 / EBL87 2	AF09_lignin	Lignin mutants and UV stress		Stem	4	44
Senescence	RIL045LNG 1 / RIL045LN 1	RA04-08_Senescence	Transcriptomic analysis of leaf senescence in Arabidopsis thaliana.	17098848	Rosette leaf	4	45
LAR	LAR_06h_T / LAR_06h_C	TRI38-LAR	Transcriptomic analysis of the localized acquired resistance phenomenon (LAR) in Arabidopsis Thaliana.		Rosette leaf	4	46
LAR	LAR_24h_T / LAR_24h_C	TRI38-LAR	Transcriptomic analysis of the localized acquired resistance phenomenon (LAR) in Arabidopsis Thaliana.		Rosette leaf	4	47
LAR	LAR_48h_T / LAR_48h_C	TRI38-LAR	Transcriptomic analysis of the localized acquired resistance phenomenon (LAR) in Arabidopsis Thaliana.		Rosette leaf	4	48
LAR	HR-6h-T / HR-6h-C	TRI38-LAR	Transcriptomic analysis of the localized acquired resistance phenomenon (LAR) in Arabidopsis Thaliana.		Rosette leaf	4	49
