# Supplementary material for: Transcriptional Regulation of Ribosome Components Are Determined by Stress According to Cellular Compartments in Arabidopsis thaliana
Source: PLoS One. 2011 Dec 2;6(12):e28070. doi: 10.1371/journal.pone.0028070 (PMC3229498; doi:10.1371/journal.pone.0028070)
Supplement: Table S6 — List of RP genes coding for mitoRP. Are provided gene reference number (AGI), and common gene names, alternative gene name are provided when it exist. Corresponding ID in CATMA and Affymetrix dataset are listed. Bibliographic evidences have been reported in the two last columns (corresponding citation number in Table S1). (PDF) [file pone.0028070.s006.pdf]

Gene reference number (AGI)	Common Gene Name	CATMA ID	Affymetrix ID	ms/ms localisation evidences	GFP localisation evidences
AT2G42710	RPL1	CATMA2A41110	263975_at		
AT2G07715	RPL2	CATMA2A07160			
AT2G44065	RPL2	CATMA2A42495	267211_at		
ATMG00560	RPL2	M164	265232_s_at		
ATMG00690	RPL2	M273	244906_at		
AT3G17465	RPL3/ RPL3P	CATMA3A16900	258404_at	19	
AT2G20060	RPL4	CATMA2A18540	265594_at		
ATMG00210	RPL5	M067	266044_s_at		
AT5G53070	RPL9	CATMA5A48970	248295_at		
AT3G12370	RPL10	CATMA3A11320	256234_at		
AT4G35490	RPL11	CATMA4A37150	253138_at		
AT1G70190	RPL12	CATMA1A59480	264702_at	19	
AT4G36420	RPL12	CATMA4A38000	246210_at		
AT4G37660	RPL12	CATMA4A39180	253058_at		
AT3G01790	RPL13	CATMA3A00800	258995_at		
AT1G17560	RPL14/ HLL	CATMA1A16610	260683_at	15	
AT5G46160	RPL14/ HLP	CATMA5A42160	248878_at		15
AT4G23620	RPL15	CATMA4A25470	254228_at		
AT5G64670	RPL15	CATMA5A60100	247249_at		
ATMG00080	RPL16	M015			
AT5G64650	RPL17	CATMA5A60080	247247_at		
AT5G09770	RPL17family	CATMA5A08580	250495_at		
AT3G22450	RPL18N	CATMA3A22450	258447_at		
AT3G45020	RPL18N	CATMA3A38040			
AT1G08845	RPL18N	CATMA1A07750			
AT1G24240	RPL19	CATMA1A23130	264874_at		
AT4G11630	RPL19				
AT4G16030	RPL19D	CATMA4A16813	245471_at		
AT3G03600	RPS2	CATMA3A02530	259196_at		
AT1G16870	RPS29	CATMA1A15860	256106_at		
ATMG00090	RPS3	M018	244944_s_at		
ATMG00290	RPS4	M097	266042_s_at	31	
AT1G64880	RPS5	CATMA1A54180	262880_at	17	
AT3G18760	RPS6	CATMA3A18370	257755_at		
AT2G07696	RPS7	CATMA2A06830			
ATMG00980	RPS7	M315	263502_s_at		
ATMG01270	RPS7		265238_s_at		
AT3G49080	RPS9	CATMA3A42090	252287_at		
AT3G22300	RPS10	CATMA3A22295	258454_at		
AT1G31817	RPS11/NFD3	CATMA1A30070	246266_at		
AT2G07679	RPS12				
AT2G07675	RPS12	CATMA2A06470			
AT1G77750	RPS13	CATMA1A66900	259678_at		20
AT2G34520	RPS14	CATMA2A32660	266955_at		
AT1G15810	RPS15		259505_at		
AT1G80620	RPS15	CATMA1A69830			
AT5G56940	RPS16	CATMA5A52690	247935_at		32
AT2G05220	RPS17B	CATMA2A04100			
AT3G10610	RPS17C	CATMA3A09650	258922_at		
AT5G04800	RPS17D		250862_s_at		
AT1G07210	RPS18		256043_at		
AT5G47320	RPS19	CATMA5A43275	248800_at		
AT1G16740	RPL20		255767_at		
AT1G57860	RPL21G		246379_s_at		
AT4G30930	RPL21/ NFD1	CATMA4A32590	253549_at		29
AT1G09690	RPL21C	CATMA1A08550	264679_s_at		
AT1G52370	RPL22	CATMA1A43430			
AT4G28360	RPL22	CATMA4A30000	253773_s_at	31	
AT1G04480	RPL23A		263665_at		
AT3G04400	RPL23C	CATMA3A03370	258569_at		
AT4G39880	RPL23N	CATMA4A41270	252850_at		
AT5G66860	RPL25	CATMA5A62290		19	
AT2G16930	RPL27		266535_s_at		
AT5G15220	RPL27				
AT5G39800	RPL27		249424_s_at		
AT1G07830	RPL29	CATMA1A06860	261418_at		
AT5G55140	RPL30	CATMA5A50910	248102_at		
AT1G07070	RPL35aA	CATMA1A06140	256065_at		
AT3G55750	RPL35aD				
AT5G40080	RPL41				
AT3G59650	RPL51/S25	CATMA3A52680	251483_at		
