# Supplementary material for: Transcriptional Regulation of Ribosome Components Are Determined by Stress According to Cellular Compartments in Arabidopsis thaliana
Source: PLoS One. 2011 Dec 2;6(12):e28070. doi: 10.1371/journal.pone.0028070 (PMC3229498; doi:10.1371/journal.pone.0028070)
Supplement: Table S7 — List of RP genes coding for plastoRP. Are provided gene reference number (AGI), and common gene names, alternative gene name are also provided when it exist. PSRP are plastid-specific RP. Corresponding ID in CATMA and Affymetrix dataset are also listed. Bibliographic localization evidences have been also summarized in the two last columns (corresponding citation number in Table S1). (PDF) [file pone.0028070.s007.pdf]

Gene reference number (AGI)	Common Gene Name	CATMA ID	Affymetrix ID	ms/ms localization evidences	GFP localization evidences
AT5G24490	PSRP-1		249742_at	9, 2	
AT3G52150	PSRP-2	CATMA3A45070	252032_at	9, 27, 6, 2, 14, 1, 15	
AT1G68590	PSRP-3		262283_at	9, 2, 14	
AT5G15760	PSRP-3	CATMA5A14030	246517_at		
AT2G38140	PSRP-4	CATMA2A36435	267088_at	9	26
AT3G56910	PSRP-5		246294_at	9, 2, 14, 1	
AT5G17870	PSRP-6	CATMA5A16150	250058_at		
AT4G29060	PSRP-7				
AT3G63490	RPL1		251120_at	11, 9, 6,13, 2	
ATCG00830	RPL2.1	C175		9	
ATCG01310	RPL2.2		244987_s_at		
AT2G43030	RPL3related	CATMA2A41430	265247_at	9, 6, 2	
AT1G07320	RPL4	CATMA1A06385	261078_at	11, 9, 6,13, 2, 14, 15	
AT4G01310	RPL5 family	CATMA4A01510	255623_at	9, 6, 2, 1	
AT1G05190	RPL6 /emb2394	CATMA1A04040	264575_at	9, 6, 2, 1	
AT3G44890	RPL9/ CL9	CATMA3A37910	246339_at	9, 6, 2	
AT5G13510	RPL10		245852_at	9, 6	
AT1G06380	RPL10a related	CATMA1A05420	259392_at	9, 1	
AT1G32990	RPL11	CATMA1A31300	261190_at	9, 6, 2, 1	
AT5G51610	RPL11	CATMA5A47540	248414_at		
AT3G27830	RPL12A			4, 9, 27,13, 2, 14	
AT3G27840	RPL12B	CATMA3A27650	257223_at		
AT3G27850	RPL12C		257225_s_at	27,13, 2, 14, 1	
AT1G78630	RPL13A	CATMA1A67690	263131_at	9, 6, 2, 1	
ATCG00780	RPL14/ HLL	C165	244982_at	9	
AT3G25920	RPL15	CATMA3A25740	258076_at	9, 6, 2	
ATCG00790	RPL16	C168	244983_at	9	
AT3G54210	RPL17	CATMA3A47150	251883_at	9	
AT3G20230	RPL18N	CATMA3A19890	257132_at		
AT5G13720	RPL18N		250247_at	9, 33	
AT1G48350	RPL18N	CATMA1A39430	262235_at	9, 1	
AT4G17560	RPL19	CATMA4A18580	245357_at	9, 1	
AT5G47190	RPL19	CATMA5A43180	248798_at	9, 1	
ATCG00660	RPL20	C139	244970_at	9	
AT1G35680	RPL21/ CL21	CATMA1A33866	262029_at	11, 9, 6, 2, 1	
ATCG00810	RPL22		244985_at	9	
ATCG00840	RPL23.1	C178		9	
ATCG01300	RPL23.2		244988_s_at		
AT5G54600	RPL24	CATMA5A50455	248174_at	9	
AT5G40950	RPL27	CATMA5A36620	249331_at	9, 6, 2, 14, 15	
AT2G33450	RPL28	CATMA2A31610	255850_at	9,	
AT5G65220	RPL29	CATMA5A60610	247201_at	9, 2, 1	
AT1G75350	RPL31	CATMA1A64680	261119_at	9, 6, 1	
ATCG01020	RPL32	C207	244960_at		
ATCG00640	RPL33	C135	244968_at	9	
AT1G29070	RPL34	CATMA1A27050	260898_at		
AT2G24090	RPL35family	CATMA2A22440	266570_at	9	
ATCG00760	RPL36	C162	244980_at	9	
AT1G71720	RPS1	CATMA1A60920	261507_at		
AT5G30510	RPS1	CATMA5A27970		9, 27, 2	
AT3G23700	RPS1	CATMA3A23650	257172_at	11, 9, 2	
ATCG00800	RPS3aN	C171	244984_at	9, 2	
ATCG00380	RPS4	C084	245009_at	9, 6	
AT2G33800	RPS5	CATMA2A32000	267435_at	11, 9, 6, 2, 14, 15	
AT1G64510	RPS6	CATMA1A53800	261954_at	9, 2, 14	

ATCG01240	RPS7.1		244992_s_at	14, 15	
ATCG00900	RPS7.2	C191		9, 2, 15	
AT1G16790	RPS8	CATMA1A15780	255759_at	9,13, 1	
ATCG00770	RPS8	C163	244981_at	9	
AT1G74970	RPS9	CATMA1A64335	262172_at	9, 6, 2, 1	
AT3G13120	RPS10	CATMA3A12140	257190_at	9	
ATCG00750	RPS11		244979_at	9	
ATCG00160	RPS12	C033	244996_at	9	
ATCG00905	RPS12	C193			
ATCG01230	RPS12		244940_at		
AT5G14320	RPS13	CATMA5A12555	250190_at	9, 2, 1	20
ATCG00330	RPS14	C071	245005_at	9	
ATCG01120	RPS15	C230	244938_at	9, 6	
ATCG00050	RPS16	C236	245049_at		
ATCG00065	RPS16	C140	244939_at		
AT4G34620	RPS16	CATMA4A36460	253201_at	9, 1	32
AT1G79850	RPS17	CATMA1A69000	260165_at	9, 6, 2, 1	
ATCG00650	RPS18	C137	244969_at	9, 6	
ATCG00820	RPS19		244986_at	9, 6	
AT3G15190	RPS20	CATMA3A14550	256855_at	9, 6, 2	
AT3G27160	RPS21 GHS1	CATMA3A26960	256753_at	2	
AT5G63300	RPS21 GHS1	CATMA5A58880			
